# Supplementary material for: Transferable deep generative modeling of intrinsically disordered protein conformations
Source: PLoS Comput Biol. 2024 May 23;20(5):e1012144. doi: 10.1371/journal.pcbi.1012144 (PMC11152266; doi:10.1371/journal.pcbi.1012144)
Supplement: S1 Text — (DOCX) [file pcbi.1012144.s001.docx]

### S1 Text. Selection of sequences.

#### Selection of the training set sequences

The training set in this study consists of 3,259 IDR sequences. The set was constructed via an incremental process involving the addition of different parts, which are described here.

1. **1089 IDRs from the training set of ABSINTH-based idpGAN.** The first part of the training set consists of data we previously collected to train the idpGAN model[29]. Two different idpGAN models were trained on either COCOMO coarse-grained simulations[13] or ABSINTH implicit solvent simulations[14]. The COCOMO-based idpGAN was trained on a set of 1,966 IDRs. These sequences originate from DisProt[63] (version 2021_06) and have lengths ranging from 20 to 200 residues. The ABSINTH-based idpGAN model was trained on a subset of these, containing all the 1089 sequences with lengths between 20 and 50 residues. This subset, for which we collected ABSINTH simulation data, is the initial part of the training set of the present study.
2. **132 IDR sequences from the training set of COCOMO-based idpGAN.** In addition to the sequences of part 1, we selected all 132 IDRs with lengths from 51 to 60 from the training set of COCOMO-based idpGAN. The goal here was to increment the maximum length of the peptides in the training set to obtain generative models able to work with longer peptides.
3. **1,888 IDR random crops from the training set of COCOMO-based idpGAN.** To further expand the training set, we added 1,888 IDR sequences with lengths ranging from 20 to 55. These sequences were generated by randomly extracting one or more continuous crops from each IDR in the training set of COCOMO-based idpGAN with a length between 56 and 200. The length of the crops was randomly sampled from a uniform distribution. In the extraction process, we ensured that no two crops taken from the same original IDR had overlapping sequences. The goal was to significantly expand the diversity of sequences with intermediary lengths in the training set.
4. **150 IDR sequence with lengths between 12 and 19.** Finally, we randomly selected from DisProt (see part 1) a new set of 150 IDRs with 12 to 19 residues. The goal was to include a portion of shorter sequences in the training set for improving modeling performance on shorter peptides.

We ran ABSINTH MCMC simulations for all IDR sequences of part 2 to 4 using the same protocol used for part 1. The resulting data was added to the data from part 1 to finally constitute the full training set of this study.

#### Selection of the validation set sequences

The validation set in this study consists of 25 peptides. To obtain their sequences, we used the Swiss-Prot database[64] containing a total of 568,744 protein sequences (retrieved on February 2023). For each sequence with at least 20 amino acids, we extracted a random crop with length between 20 and 55 (uniformly sampled). We then filtered the sequences via simple and approximate criteria for IDP classification based on charge-hydropathy values[65]. We kept all crops classified a disordered and a random 20% fraction of the crops classified as not disordered. From all these sequences, we randomly selected 25 for constituting the validation set. For these peptides, we ran MCMC simulations using the same protocol employed for the training set.
