# Supplementary material for: Transferable deep generative modeling of intrinsically disordered protein conformations
Source: PLoS Comput Biol. 2024 May 23;20(5):e1012144. doi: 10.1371/journal.pcbi.1012144 (PMC11152266; doi:10.1371/journal.pcbi.1012144)
Supplement: S2 Text — (DOCX) [file pcbi.1012144.s002.docx]

### S2 Text. MCMC simulation protocol.

Here we describe the protocols used to run MCMC simulations via CAMPARI[11] in this study, which was adopted from a previous study for simulating charged IDRs[58].

#### MCMC runs at 298 K

**Hamiltonian**: simulations were performed using the OPLS-AA/L force field and the ABSINTH implicit solvent model. The CAMPARI parameter file *abs3.1_opls.prm* was used. Cutoffs for Lennard-Jones and electrostatic interactions were set at 10 and 14 Å respectively.

**Peptide modeling**: peptides were capped with an acetyl group at the N-terminus and a N-methylamide group at the C-terminus. Histidine sidechains were protonated only at the ε position, making them neutrally charged.

**System**: peptides were placed inside a spherical droplet. The radius of the droplet was set to 70 Å for peptides with *L* <= 34 residues (“short” peptides) and 100 Å for peptides with *L* >= 35 residues (“long” peptides). Na^+^ and Cl^-^ ions were added to neutralize net peptide charges and to represent a 125 mM salt solution (resulting in 108 excess ion pairs for 70 Å droplets and 315 pairs for 100 Å droplets).

**Sampling**: Metropolis MCMC simulations were performed in the NVT ensemble at 298 K. The degrees of freedom in the simulations were backbone φ, ψ, ω torsion angles and sidechain χ torsion angles and rigid-body coordinates for peptides molecules and ions.

**Monte Carlo move set**: the move set was based on a one employed previously[58].

**Simulations**: independent simulations were performed using randomly generated initial conformations. For “short” peptides, we performed 1 × 10^6^ equilibration and 2.5 × 10^7^ production steps. For “long” peptides, we performed 2 × 10^6^ equilibration and 5 × 10^7^ production steps.

**Output**: snapshots were saved every 5,000 steps during the production phase, resulting in 5,000 and 10,000 snapshots for a “short” and “long” peptide simulation respectively.

#### Replica exchange (RE) simulations

For four test set peptides (nls, protan, protac and drk_sh3), we additionally performed thermal RE MCMC simulations as implemented in CAMPARI. The RE strategy was inspired by one used previously to simulate polyampholytic peptides[30]. The temperature schedule comprised at least 12 temperatures: [298K, 302K, 306K, 310K, 315K, 320K, 330K, 340K, 350K, 360K, 370K, 380K]. For all simulations of nls and drk_sh3 and a small portion of protan and protac simulations, we added 4 temperatures: [390K ,400K, 410K, 420K]. The simulation setup (Hamiltonian, system preparation and MCMC sampling) was the same described above for simulations at constant 298 K. Each thermal replica was initiated using randomly generated initial conformations. For most RE simulations, we performed 5 × 10^5^ equilibration and 1.25 × 10^7^ production steps. In a minority of cases, we performed 1 × 10^6^ equilibration and 2.5 × 10^7^ production steps. Swaps between two neighboring replicas were always attempted every 2,000 steps. Snapshots were saved every 5,000 steps.
