# Supplementary material for: Transferable deep generative modeling of intrinsically disordered protein conformations
Source: PLoS Comput Biol. 2024 May 23;20(5):e1012144. doi: 10.1371/journal.pcbi.1012144 (PMC11152266; doi:10.1371/journal.pcbi.1012144)
Supplement: S3 Text — [83,84,85]. (DOCX) [file pcbi.1012144.s003.docx]

### S3 Text. Neural networks of idpSAM.

This section describes the neural networks of idpSAM with pseudocode for some key operations. The PyTorch code for all networks can be found at: <https://github.com/giacomo-janson/idpsam> [83]

#### Encoder

The encoder network $E_{\phi}$ of is based on a transformer architecture (**S18 Fig A** and **B**). It has 912,888 trainable parameters.

**Input***.* The input of $E_{\phi}$ consist of: (i) two types of geometrical features extracted from the coordinates $\mathbf{x}\in\mathbb{R}^{L\times3}$ of the Cα atoms of a peptide ($L$ is the number of its residues); (ii) the amino acid sequence tokens of the peptide $\mathbf{a}\in\mathbb{R}^{L\times1}$. The first type of geometrical features consists in the full distance matrix $\mathbf{d}\in\mathbb{R}^{L\times L}$ calculated from $\mathbf{x}$. This is processed by a radial base function (RBF) expansion as in SchNet[61, 77] with 320 equally spaced Gaussians from 0.0 to 30.0 Å. The RBF expansion is embedded to a dimension of $c_{dist}=192$ by a multilayer perceptron (MLP) with a GELU activation[84]:

$\mathbf{h}_{dist}=\mathrm{Linear}(\mathrm{GELU}(\mathrm{Linear}(\mathrm{RBF}(\mathbf{d}))))$ $\mathbf{h}_{dist}\in\mathbb{R}^{L\times L\times c_{dist}}$

The second type of geometrical features is a sequence $\mathbf{t}\in\mathbb{R}^{L\times3}$ storing α torsion angle values (see main text). The first two channels of $\mathbf{t}$ store the cosine and sine values of the $L-3$ angles of a peptide, with the first and last two positions padded with 0. The third channel contains a mask with values of 0 for the three zero-padded positions and 1 for the rest. These features are embedded to a dimension of $c_{node}=128$ by an MLP:

$\mathbf{h}_{tors}=\mathrm{Linear}(\mathrm{GELU}(\mathrm{Linear}(\mathbf{t})))$ $\mathbf{h}_{tors}\in\mathbb{R}^{L\times c_{node}}$

The amino acid sequence $\mathbf{a}$ is processed via a learnable embedding layer[4, 29] to yield an amino acid embedding $\mathbf{h}_{aa}\in\mathbb{R}^{L\times c_{aa}}$ (with $c_{aa}=32$).

**Transformer blocks***.* The network has $n_{blocks}=5$ transformer blocks[36] with self-attention and $n_{heads}=8$ heads, a “pre” layer normalization configuration[85], an MLP with hidden dimension of 256, GELU activation and no dropout. The input of the first block is$\mathbf{h}^{[1]}=\mathbf{h}_{tors}$. At each block $l$, a linear layer prepares the input of the block in the following way:

$\mathbf{h}_{in}^{[l]}=\mathrm{Linear}(\mathrm{concat}(\mathbf{h}^{[l]},\mathbf{h}_{aa}))$ $\mathbf{h}^{[l]}\in\mathbb{R}^{L\times c_{node}}, \mathbf{h}_{in}^{[l]}\in\mathbb{R}^{L\times c_{node}}$

where$\mathbf{h}^{[l]}$ is the input sequence at block $l$.

Also, at every block the distance features $\mathbf{h}_{dist}$ are concatenated to a learnable 2d relative positional embedding $\mathbf{h}_{pos2d}\in\mathbb{R}^{L\times L\times c_{pos2d}}$ (with $c_{pos2d}=64$) used in AlphaFold2 and other model[4, 29]. These concatenated features are projected via a linear layer to obtain:

$\mathbf{h}_{bias}^{[l]}=\mathrm{Linear}(\mathrm{concat}(\mathbf{h}_{dist},\mathbf{h}_{pos2d}))$ $\mathbf{h}_{bias}^{[l]}\in\mathbb{R}^{L\times L\times n_{heads}}$

which contains bias terms that are then added to the logit values of the attention heads in the layer. In summary, the transformer block operates the following update:

$\mathbf{h}^{[l+1]}=\mathrm{Transformer}(input=\mathbf{h}_{in}^{[l]},bias=\mathbf{h}_{bias}^{[l]})$

**Output**. The output of the last layer if processed via:

$\mathbf{z}=\mathrm{LayerNorm}\left( \mathrm{Linear}(\mathrm{GELU}(\mathrm{Linear}(\mathbf{h}^{\left[ n_{blocks} \right]})),out\_dim=c) \right)$ $\mathbf{z}\in\mathbb{R}^{L\times c}$

where $z$ is the encoded representation of the Cα coordinates of the peptide and $c=16$. The layer normalization operation does not use learnable elementwise affine parameters. It is used to rescale the output of the encoder to help downstream applications with other neural networks.

#### Scaling of interatomic distances in the AE loss

The AE loss used in this study includes a term for the reconstruction of Cα-Cα interatomic distances. Before calculating the loss, a distance $d_{ij}=\|\mathbf{x}_{i}-\mathbf{x}_{j}\|$ between the Cα atoms of residue $i$ and $j$ with sequence separation $k=\left| i-j \right|$ is first scaled by:

$$s\left( d_{ij},k \right)=\frac{{(d}_{ij}-m_{k})}{s_{k}}$$

where $m_{k}$ and $s_{k}$ are the mean and standard deviation in the AE training set for Cα-Cα distances with sequence separation $k$. We found this standardization procedure to be critical for efficiently training the AE model, since the scale of distances between atom pairs with different $k$ values can vary greatly.

#### Decoder

The decoder $D_{\psi}$ of SAM is similar to the encoder (**S18 Fig C**). It has 1,161,347 trainable parameters. In describing its components, we employ similar notations to those used for the encoder, for reasons of economy and clarity.

**Input**. The input of the decoder is an encoding $\mathbf{z}$. This is first projected to a dimension of $c_{node}=128$ by an MLP:

$\mathbf{h}_{enc}=\mathrm{Linear}(\mathrm{GELU}(\mathrm{Linear}(\mathbf{z})))$ $\mathbf{h}_{enc}\in\mathbb{R}^{L\times c_{node}}$

which is the input to the first transformer block, so $\mathbf{h}^{[1]}=\mathbf{h}_{enc}$.

**Transformer blocks**. The network has a stack of $n_{blocks}=5$ modified transformer blocks. They share similar architecture and hyper-parameters of the blocks in $E_{\phi}$, with only one exception: instead of using a scaled dot product attention[36] with 8 heads, they use $n_{heads}=32$ heads with an attention mechanism inspired by the kernel self-attention in the Timewarp model[28] and AF2 invariant point attention[4]. More specifically, the input $\mathbf{h}^{[l]}$ of a block $l$ is first mapped to query and key 3D coordinates:

$\mathbf{q}^{(m)}=\mathrm{Linear}\left( \mathbf{h}^{\left[ l \right]} \right)$ $\mathbf{q}^{(m)}\in\mathbb{R}^{L\times3}$

$\mathbf{k}^{(m)}=\mathrm{Linear}(\mathbf{h}^{[l]})$ $\mathbf{k}^{(m)}\in\mathbb{R}^{L\times3}$

where $m$ is the index of the head (we omit the block index in the notations for $\mathbf{q}$ and $\mathbf{k}$). Value vectors are instead obtained with the usual mechanism used in dot product attention. The logit values $y_{ij}^{(m)}$ of the attention map are calculated by:

$y_{ij}^{(m)}=-\frac{{\|\mathbf{q}_{i}^{(m)}-\mathbf{k}_{j}^{(m)}\|}^{2}}{l^{(m)}}$ $y_{ij}^{(m)}\in\mathbb{R}$

with:

$$l^{(m)}=\mathrm{softplus}(\lambda^{\left( m \right)}+\epsilon)$$

where $\lambda^{\left( m \right)}$ is a learnable scaling parameter and $\epsilon$is a small real number for numerical stability. Similar to the encoder, the decoder uses a layer for creating 2d relative positional embeddings with $c_{pos2d}=64$. These are linearly projected to obtain bias values $\mathbf{h}_{bias}^{[l]}\in\mathbb{R}^{L\times L\times n_{heads}}$ which are summed to logits to obtain final attention maps. Like in dot product attention, the maps are then used to update $\mathbf{h}^{\left[ l \right]}$ by matrix multiplication with the values vectors. Overall, the update is:

$$\mathbf{h}^{[l+1]}=\mathrm{ModifiedTransformer}(input=\mathbf{h}^{[l]},bias=\mathbf{h}_{bias}^{[l]})$$

We found the use of this self-attention mechanism to slightly improve the reconstruction accuracy of the decoder. We hypothesize that the improvement could be caused by better inductive biases for modeling Cartesian coordinates.

**Output**. The output of the final transformer block is ultimately mapped to a tensor $\tilde{\mathbf{x}}\in\mathbb{R}^{L\times3}$ by an MLP:

$$\tilde{\mathbf{x}}=\mathrm{Linear}(\mathrm{GELU}(\mathrm{Linear}(\mathbf{h}^{\left[ n_{blocks} \right]})),out\_dim=3)$$

which represent the reconstructed Cα coordinates of an encoding $\mathbf{z}$.

#### Noise prediction network

The noise prediction network $\boldsymbol{\epsilon}_{\theta}$ of SAM is also based on a transformer architecture (**S19 Fig**). It has 15,161,296 trainable parameters. In describing its components, we again employ similar notations to those used for the other networks.

**Input***.* The input of $\boldsymbol{\epsilon}_{\theta}$ consist of: (i) an encoded peptide conformation $\mathbf{z}_{t}\in\mathbb{R}^{L\times c}$perturbed at an integer-valued timestep $1<t\leq T$; (ii) the timestep $t$; (iii) the amino acid sequence $\mathbf{a}$ of the peptide. The input encoding is first project to the node hidden dimension $c_{node}=256$ by a linear layer:

$\mathbf{h}_{enc}=\mathrm{Linear}\left( \mathbf{z}_{t} \right)$ $\mathbf{h}_{enc}\in\mathbb{R}^{L\times c_{node}}$

which is the input to the first transformer block, so $\mathbf{h}^{[1]}=\mathbf{h}_{enc}$. The input timestep $t$ is processed via a sinusoidal embedding and an MLP with a SiLU activation to project it to a dimension of $c_{time}=256$, then is finally tiled to a sequence of $L$ tokens:

$\mathbf{h}_{time}=\mathrm{Linear}\left( \mathrm{SiLU}(\mathrm{Linear}(\mathrm{SinusoidalEmbed}(t,dim=256)) \right)$ $\mathbf{h}_{time}\in\mathbb{R}^{1\times c_{time}}$

$\mathbf{h}_{time}\leftarrow\mathrm{tile}(\mathbf{h}_{time},dim=0)$ $\mathbf{h}_{time}\in\mathbb{R}^{L\times c_{time}}$

Similar to the encoder, $\boldsymbol{\epsilon}_{\theta}$ uses a layer for creating amino acid embedding $\mathbf{h}_{aa}\in\mathbb{R}^{L\times c_{aa}}$ (with $c_{aa}=32$).

**Transformer blocks***.* The network has $n_{blocks}=16$ modified transformer blocks with self-attention with $n_{heads}=16$ heads, a “pre” layer normalization configuration, an MLP with hidden dimension of 512, GELU activation and no dropout. The modification consists in the way timestep and amino acid embeddings are injected. We use the adaLN-Zero (LN: layer normalization) mechanism from the Latent Diffusion Transformer[31]. The scale ($\boldsymbol{\gamma}$), shift ($\boldsymbol{\beta}$) and gate ($\boldsymbol{\alpha}$) values for adaLN-Zero are obtained by:

$\mathbf{c}_{in}=\mathbf{h}_{time}+\mathrm{Linear}\left( \mathbf{h}_{aa} \right)$ $\mathbf{c}_{in}\in\mathbb{R}^{L\times c_{time}}$

$\boldsymbol{\gamma}_{1},\boldsymbol{\beta}_{1},\boldsymbol{\alpha}_{1},\boldsymbol{\gamma}_{2},\boldsymbol{\beta}_{2},\boldsymbol{\alpha}_{2}=\mathrm{Linear}\left( \mathrm{GELU}\left( \mathbf{c}_{in} \right) \right)$ $\boldsymbol{\gamma}_{1},\boldsymbol{\beta}_{1},\boldsymbol{\alpha}_{1},\boldsymbol{\gamma}_{2},\boldsymbol{\beta}_{2},\boldsymbol{\alpha}_{2}\in\mathbb{R}^{L\times c_{node}}$

$$\boldsymbol{\zeta}_{in}=(\boldsymbol{\gamma}_{1},\boldsymbol{\beta}_{1},\boldsymbol{\alpha}_{1},\boldsymbol{\gamma}_{2},\boldsymbol{\beta}_{2},\boldsymbol{\alpha}_{2})$$

these values are used to normalize the embedded sequence in the transformer block (we omit the block index in their notations). We found the use of adaLN-Zero mechanism highly beneficial for the performance of SAM (see the main text), which is consistent with Peebles and Xie[31]. The injection mechanism of the adaLN-Zero we implement is illustrated in **S19 Fig B** and the gate and modulate algorithms are given below (the $⨀$ operator indicates element-wise multiplication and addition with 1 involves broadcasting):

$\mathbf{def} \mathrm{modulate}\left( \mathbf{h}\in\mathbb{R}^{L\times c_{node}}, \boldsymbol{\gamma}\in\mathbb{R}^{L\times c_{node}},\boldsymbol{\beta}\in\mathbb{R}^{L\times c_{node}} \right):$

$$\mathbf{return} \mathbf{h} ⨀ (1+\boldsymbol{\gamma})+ \boldsymbol{\beta}$$

$\mathbf{def} \mathrm{gate}\left( \mathbf{h}\in\mathbb{R}^{L\times c_{node}},\boldsymbol{\alpha}\in\mathbb{R}^{L\times c_{node}} \right):$

$$\mathbf{return} \mathbf{h} ⨀ \boldsymbol{\alpha}$$

Note that the layer normalization operations preceding the adaLN-Zero operations do not use learnable element wise affine parameters. Similar to both AE networks, $\boldsymbol{\varepsilon}_{\theta}$ also uses a layer for creating 2D relative positional embeddings with $c_{pos2d}=64$. Again, these are linearly projected to bias values $\mathbf{h}_{bias}^{[l]}\in\mathbb{R}^{L\times L\times n_{heads}}$ which are summed to logits to obtain attention maps for a regular multi-head self-attention mechanism. The modified transformer block update can be summarized as:

$$\mathbf{h}_{out}^{[l]}=\mathrm{LatentDiffusionTransformer}(input=\mathbf{h}^{\left[ l \right]},bias=\mathbf{h}_{bias}^{\left[ l \right]},adalnzero\_input=\boldsymbol{\zeta}_{in}^{\left[ l \right]})$$

to complete the update, the input of the first transformer block is used to carry out the following operation at every block:

$$\mathbf{h}^{\left[ l+1 \right]}=\mathrm{LayerNorm}(\mathbf{h}_{out}^{\left[ l \right]}+\mathrm{Linear}(\mathbf{h}^{\left[ 1 \right]}))$$

We found the injection of the input embedding at every block to be beneficial for SAM performance.

**Output**. The output of the last block if processed via:

$\hat{\boldsymbol{\epsilon}}=\mathrm{Linear}(\mathrm{GELU}(\mathrm{Linear}(\mathbf{h}^{\left[ n_{blocks} \right]})),out\_dim=c)$ $\hat{\boldsymbol{\epsilon}}\in\mathbb{R}^{L\times c}$

where $\hat{\boldsymbol{\epsilon}}$ is the predicted noise.

#### Standardization of encodings

To numerically help DDPM training, each of the $c$ channels in a training set encoding is normalized via a standard scaler using the mean and standard deviation of the channel in the dataset. At inference time, the encodings generated by the DDPM are first transformed back by the inverse of the standard scalar function. Then they are used as input for the decoder. Note that the encodings produced by $E_{\phi}$ are already normalized via a layer normalization operation in the output module (see above), but we found this additional standardization procedure to slightly improve the training stability of the DDPM.
