# Supplementary material for: Transferable deep generative modeling of intrinsically disordered protein conformations
Source: PLoS Comput Biol. 2024 May 23;20(5):e1012144. doi: 10.1371/journal.pcbi.1012144 (PMC11152266; doi:10.1371/journal.pcbi.1012144)
Supplement: S4 Text — (DOCX) [file pcbi.1012144.s004.docx]

### S4 Text. Biased latent diffusion sampling.

This section describes biased diffusion for the latent DDPM of idpSAM. We use the technique to guide idpSAM sampling towards a target radius-of-gyration (*R*_g_). The method is based on the “diffusion with external potentials” approach presented in RFDiffusion[25]. We adapted it to work in a latent diffusion model operating on encodings of 3D structures. The default reverse diffusion process of our DDPM is based on the following update rule[19]:

$\mathbf{z}_{t-1}=a_{t}\mathbf{z}_{t}-b_{t}\boldsymbol{\epsilon}_{\theta}(\mathbf{z}_{t},t,\mathbf{a}) + c_{t}\mathbf{w}$ (1)

where $\mathbf{z}_{t}$ and $\mathbf{z}_{t-1}$are encodings at time $t$ and $t-1$, $\boldsymbol{\epsilon}_{\theta}(\mathbf{z}_{t},t,\mathbf{a})$ is the output of the noise prediction, $\boldsymbol{w\sim}\mathcal{N}(\boldsymbol{0},\mathbf{I})$ is Gaussian noise and $a_{t}$, $b_{t}$ and $c_{t}$ are scalar values, functions of time $t$ which we denote here in a simplified way with respect to the original notation[19]. In biased diffusion sampling, we maintain the same update rule, but modify the prediction of $\boldsymbol{\epsilon}_{\theta}$ with a biasing term. Specifically, we substitute the output of $\boldsymbol{\epsilon}_{\theta}$ with ${\hat{\boldsymbol{\epsilon}}}_{\theta}$ via the rule:

${\hat{\boldsymbol{\epsilon}}}_{\theta}(\mathbf{z}_{t},t,\mathbf{a})=\left\{ \begin{aligned} \boldsymbol{\epsilon}_{\theta}(\mathbf{z}_{t},t,\mathbf{a}), & \mathrm{if} t/T>0.35 \\ \boldsymbol{\epsilon}_{\theta}(\mathbf{z}_{t},t,\mathbf{a})+\left( \frac{T-t}{T} \right)\mathbf{b}(\mathbf{z}_{t}), & \mathrm{if} t/T\leq0.35 \end{aligned} \right.$ (2)

where $T=1,000$ is the total number of diffusion steps, $\mathbf{b}(\mathbf{z}_{t})$ is the bias term. Using this rule, we apply regular diffusion for the first 65% steps of the diffusion process and apply biased diffusion in the last 35%. We scale the bias contribution by a factor of $\left( \frac{T-t}{T} \right)$ so that the bias becomes stronger towards the end of the diffusion process when the final structures are almost formed. The biasing term is:

$\mathbf{b}(\mathbf{z}_{t})=\nabla_{\mathbf{z}_{t}}\left( U_{\mathrm{bias}}(D_{\psi}\left( \mathbf{z}_{t} \right)) \right)$ (3)

where $D_{\psi}\left( \mathbf{z}_{t} \right)={\tilde{\mathbf{x}}}_{t}$ is a decoded 3D structure of a Cα protein conformation at time $t$ obtained by the decoder network $D_{\psi}$ of idpSAM. $U_{\mathrm{bias}}$ is the biasing potential, a differentiable scalar function taking as input a 3D structure. Note that $U_{\mathrm{bias}}$ can be differentiated with respect to an encoding $\mathbf{z}_{t}$, because the decoder network $D_{\psi}$ is also a differentiable function, so the gradient of $U_{\mathrm{bias}}$ with respect to $\mathbf{z}_{t}$ can be obtained by the chain rule (**S20 Fig**). The decoder is necessary to convert a latent representation $\mathbf{z}_{t}$ to an actual 3D structure, so that a sample a time $t$ can be scored with a $U_{\mathrm{bias}}$function that takes Cartesian features into account, such as distances or *R*_g_. The biasing gradient is employed to guide latent sampling in a direction that tends to minimize $U_{\mathrm{bias}}$. The biasing potential is:

$U_{bias}({\tilde{\mathbf{x}}}_{t})={w_{\mathrm{rg}}\left( R_{g}\left( {\tilde{\mathbf{x}}}_{t} \right)-\rho_{\mathrm{bias}} \right)}^{2}+w_{\mathrm{bl}}U_{\mathrm{bl}}({\tilde{\mathbf{x}}}_{t};l_{0})+w_{\mathrm{excl}}U_{\mathrm{excl}}({\tilde{\mathbf{x}}}_{t};d_{\min})$ (4)

where $R_{g}\left( {\tilde{\mathbf{x}}}_{t} \right)$ is the radius-of-gyration of a conformation ${\tilde{\mathbf{x}}}_{t}$ and $\rho_{\mathrm{bias}}$ is the target *R*_g_ value towards which we want to guide sampling. In our experiments, set $\rho_{\mathrm{bias}}$ as the value of the experimental *R*_g_ (**S5 Table**) minus $0.1 \mathrm{nm}$, to account for the fact that idpSAM operates on Cα atoms only. This is because the difference between the *R*_g_ of an all-atom protein structure and its Cα trace is roughly $0.1 \mathrm{nm}$. $U_{\mathrm{bl}}$ is a bond length potential which imposes an harmonic potential on all pairs of Cα atoms of adjacent residues with equilibrium length $l_{0}=0.38 \mathrm{nm}$. Its role is to help maintain chain connectivity. $U_{\mathrm{excl}}$ is an half-harmonic potential centered at $d_{\min}=0.4 \mathrm{nm}$ acting on Cα-Cα distances of residues with sequence separation > 2. Its role is to help prevent clashes, since it acts only when a distance is < $d_{\min}$. We found the addition of $U_{\mathrm{bl}}$ and $U_{\mathrm{excl}}$ to stabilize biased latent diffusion. Finally, $w_{\mathrm{rg}}=125.0$, $w_{\mathrm{bl}}=5.0$ and $w_{\mathrm{excl}}=5.0$ are weights whose values we identified empirically.
