# Supplementary material for: Transferable deep generative modeling of intrinsically disordered protein conformations
Source: PLoS Comput Biol. 2024 May 23;20(5):e1012144. doi: 10.1371/journal.pcbi.1012144 (PMC11152266; doi:10.1371/journal.pcbi.1012144)
Supplement: S5 Text — (DOCX) [file pcbi.1012144.s005.docx]

### S5 Text. Evaluation metrics.

For comparing a proposed structural ensemble (e.g.: from SAM) of a peptide with a reference MCMC ensemble of the same peptide, we used different evaluation scores. In this work, they are calculated using ensembles with 10,000 conformations each.

#### MSE_c

## The Cα-Cα contact probabilities for a peptide of length $L$ were evaluated with the following mean squared error (MSE) score:

$MSE\_c=\frac{1}{N_{pairs}}\sum_{j-i>1} \left( log\left( p_{ij} \right)-log\left( \hat{p}_{ij} \right) \right)^{2}$, (1)

where $N_{pairs}=(L-1) \times(L-2)/2$ is the number of residue pairs with sequence separation > 1 and $p_{ij}$ and $\hat{p}_{ij}$ are the contact frequencies for residues $i$ and $j$ in the reference and generated ensembles respectively. To avoid frequencies equal to zero, we use a pseudo-count value of 0.01. Contacts are defined with a Cα distance threshold of 8.0 Å.

In contrast to a similar score, we used previously, this score only considers pairs of residues with a sequence separation of at least 2, since there is very little variability in the distance between two adjacent Cα atoms and predicting contact frequencies would be trivial. This modification is included in the remaining scores considering a pair of residues (MSE_d, aKLD_d and aJSD_d).

#### MSE_d

To evaluate average Cα-Cα interatomic distance values, we use the following MSE score:

$MSE\_d=\frac{1}{N_{pairs}}\sum_{j-i>1} \left( m_{ij}-\hat{m}_{ij} \right)^{2}$, (2)

where $m_{ij}$ and $\hat{m}_{ij}$ are the average distances between the Cα atoms of residue $i$ and $j$ in the reference and generated ensembles.

#### KLD approximations

To compare mono-dimensional distributions of continuous features, we employ an approximation of the Kullback-Leibler divergence (KLD) by binning the values of the features as we did similarly to previous work^3^. We first take the minimum and maximum value of a feature over the reference and generated ensembles and split the range in $N_{bins}=50$ equally-spaced bins. We then approximate KLD as:

$\mathrm{KLD}(P\parallel Q)=\sum_{k}^{N_{bins}} P_{k}\frac{log(P_{k})}{log(Q_{k})}$, (3)

where $k$ is the index of a bin and $P_{k}$ and $Q_{k}$ are the frequencies calculated in bin $k$ (using a pseudo-count value of 0.001) for the reference and generated ensembles, respectively.

#### aKLD_d

To compare pairwise Cα-Cα interatomic distance distributions, we use the aKLD_d score, which is computed as:

$aKLD\_d= \frac{1}{N_{pairs}}\sum_{j-i>1} KLD(M_{ij}\parallel\hat{M}_{ij})$, (4)

where $M_{ij}$ and $\hat{M}_{ij}$ are the distributions of Cα-Cα distances between residue $i$ and $j$ in the reference and generated ensembles.

#### aKLD_t

To compare distribution of α torsion angles, we use the aKLD_t score, which is computed as:

$aKLD\_t= \frac{1}{N_{torsion}}\sum_{i} \mathrm{KLD}(A_{i,i+1,i+2,i+3}\parallel\hat{A}_{i,i+1,i+2,i+3})$, (5)

where $N_{torsion}=L-3$ is the number α angles in a peptide and where $A_{i,i+1,i+2,i+3}$ and $\hat{A}_{i,i+1,i+2,i+3}$ are the distributions of α angles among residue $i$ and its next 3 residues in the reference and generated ensembles.

#### KLD_r

To compare Cα radius-of-gyration distributions, we use the the KLD_r score:

$KLD\_r=\mathrm{KLD}(R\parallel\hat{R})$, (6)

where $R$ and $\hat{R}$ are the radius-of-gyration distributions for the reference and generated ensembles.

#### aJSD_d and aJSD_t

The KLD values that we use in the scores above are asymmetric, since swapping the reference and proposed distributions would result in a different KLD value. Therefore, KLD-based scores assume that there is a reference distribution. To compare pairs of distributions $P$ and $Q$ in which we do not assume any of them to be a reference, we use the symmetric Jensen-Shannon (JS) divergence which we compute via the KLD approximation above:

$\mathrm{JSD}=\frac{1}{2}\mathrm{KLD}\left( P\parallel M \right)+\frac{1}{2}\mathrm{KLD}\left( Q\parallel M \right)$, (7)

where $M$ is a mixture distribution for which we compute frequencies as:

$M_{k}=\frac{1}{2}P_{k}+\frac{1}{2}Q_{k}.$ (8)

The aJSD_d and aJSD_t scores that we use to compare some ensembles in this work are calculated similarly to the aKLD_d and aKLD_t scores defined above, but instead use this JSD approximation.
