# Supplementary figures and images for: Transferable deep generative modeling of intrinsically disordered protein conformations
Source: PLoS Comput Biol. 2024 May 23;20(5):e1012144. doi: 10.1371/journal.pcbi.1012144 (PMC11152266; doi:10.1371/journal.pcbi.1012144)

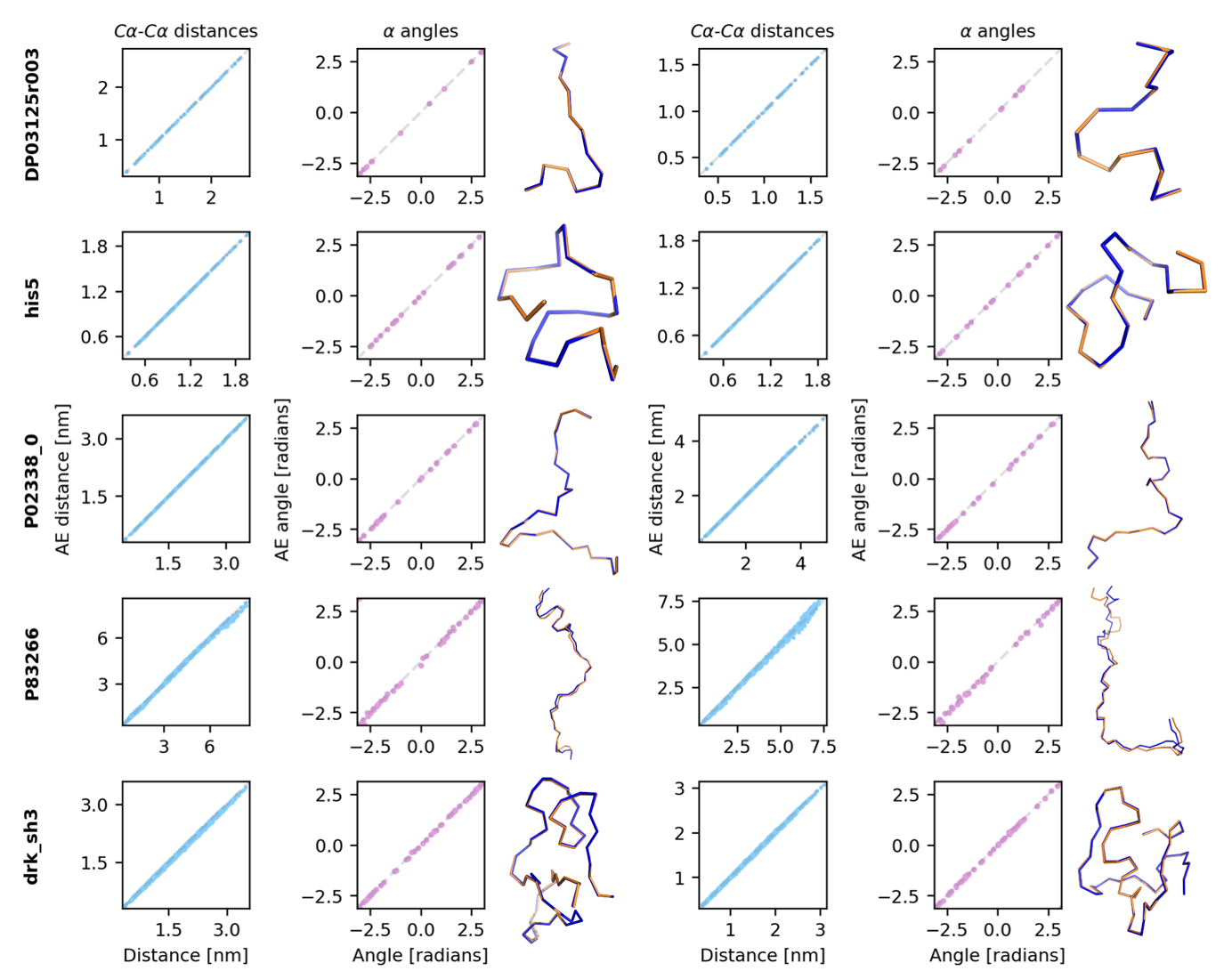

Supplement: S1 Fig — Each row shows two MCMC conformations randomly extracted from simulation data of a test peptide (only five peptides are shown). Conformations were encoded and decoded back by the AE. For each conformation, we show: (i) on the left, a scatter plot with the original Cα-Cα distances against the corresponding values in the AE reconstruction; (ii) on the middle, a scatterplot with the original α angles against the reconstructed values; (iii) on the right, a superposition of the original (blue) and reconstructed (orange) Cα structures. In the scatterplots, original values are on horizontal axes, reconstructed values on vertical axes. Examples were randomly selected. (TIF) [file pcbi.1012144.s006.tif]

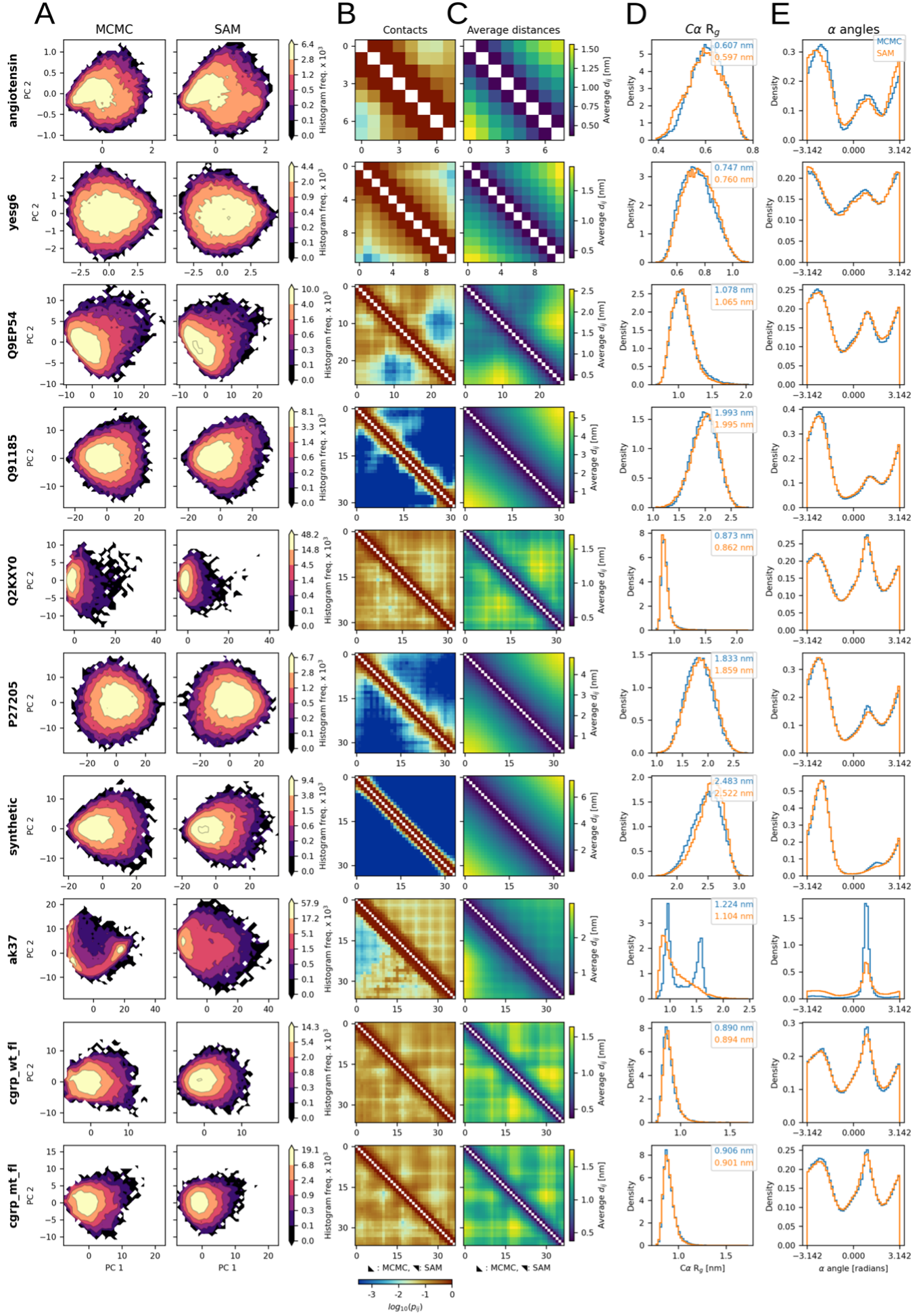

Supplement: S2 Fig — Each row shows the ensembles of a peptide from the test set: angiotensin (L = 8), yesg6 (L = 12), Q9EP54 (L = 27), Q91185 (L = 32), Q2KXY0 (L = 33), P27205 (L = 34), synthetic (L = 34), ak37 (L = 37), cgrp_wt_fl (L = 37) and cgrp_mt_fl (L = 37). See Fig 2 in the main text for more details. (TIF) [file pcbi.1012144.s007.tif]

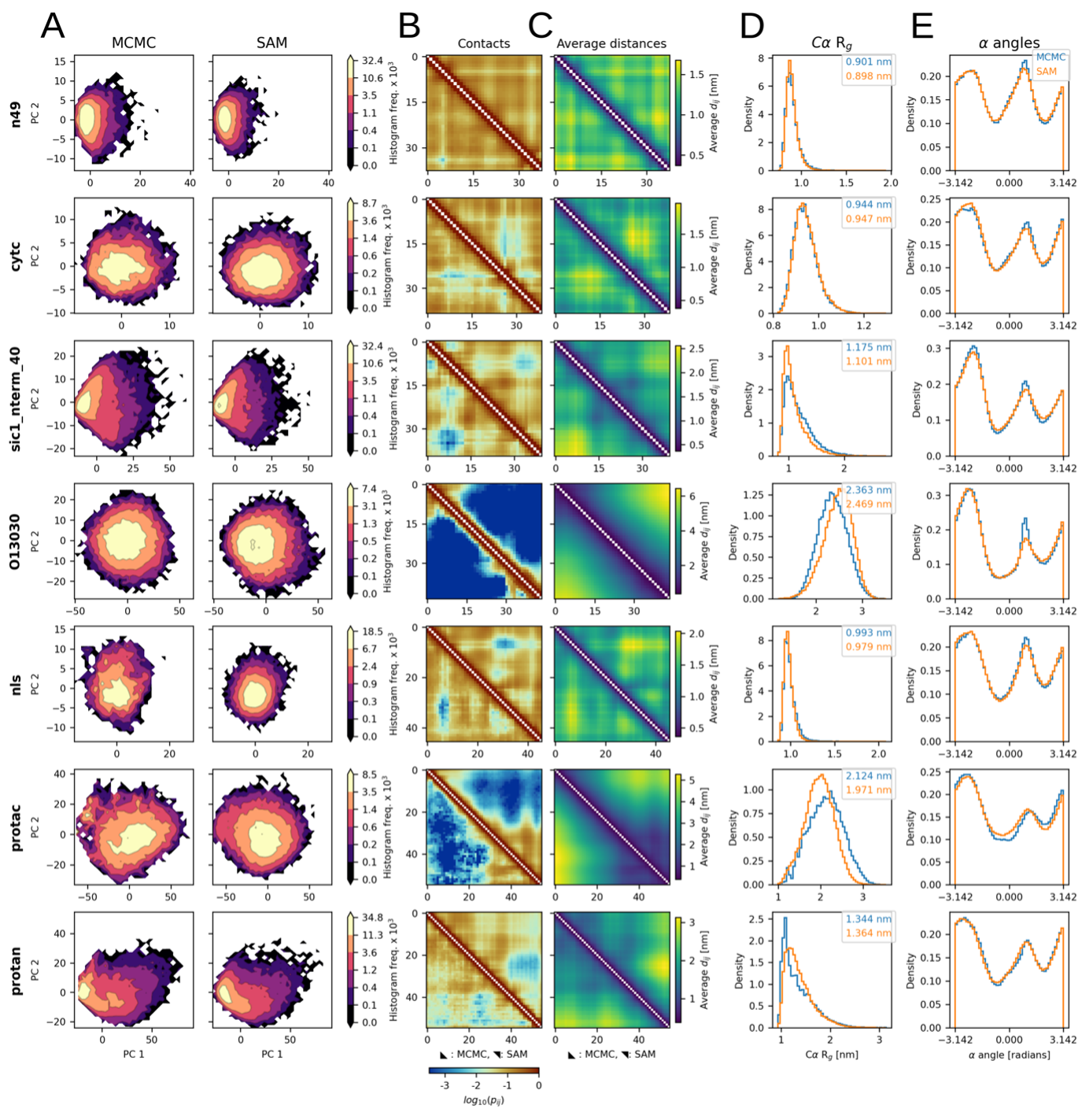

Supplement: S3 Fig — Each row shows the ensembles of a peptide from the test set: n49 (L = 38), cytc (L = 39), sic1_nterm_40 (L = 40), O13030 (L = 44), nls (L = 46), protac (L = 55) and protan (L = 55). See Fig 2 for more details. (TIF) [file pcbi.1012144.s008.tif]

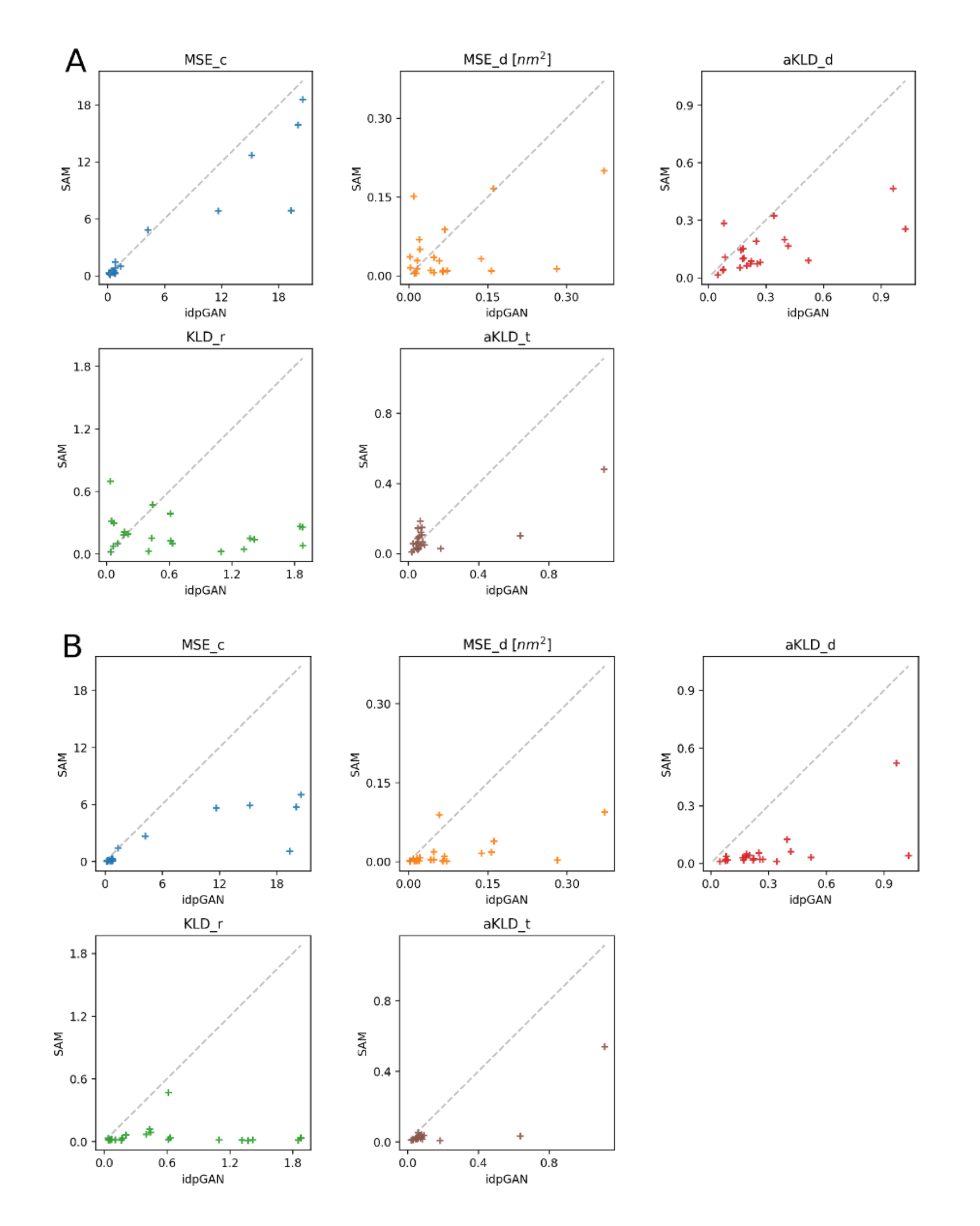

Supplement: S4 Fig — (A) Each subplot confronts the evaluation scores of the two models for the 22 test set peptides. The SAM version evaluated here was trained with the same dataset of idpGAN. (B) Similar confrontations, but the SAM version evaluated here was trained with the entire training set presented in this study. (TIF) [file pcbi.1012144.s009.tif]

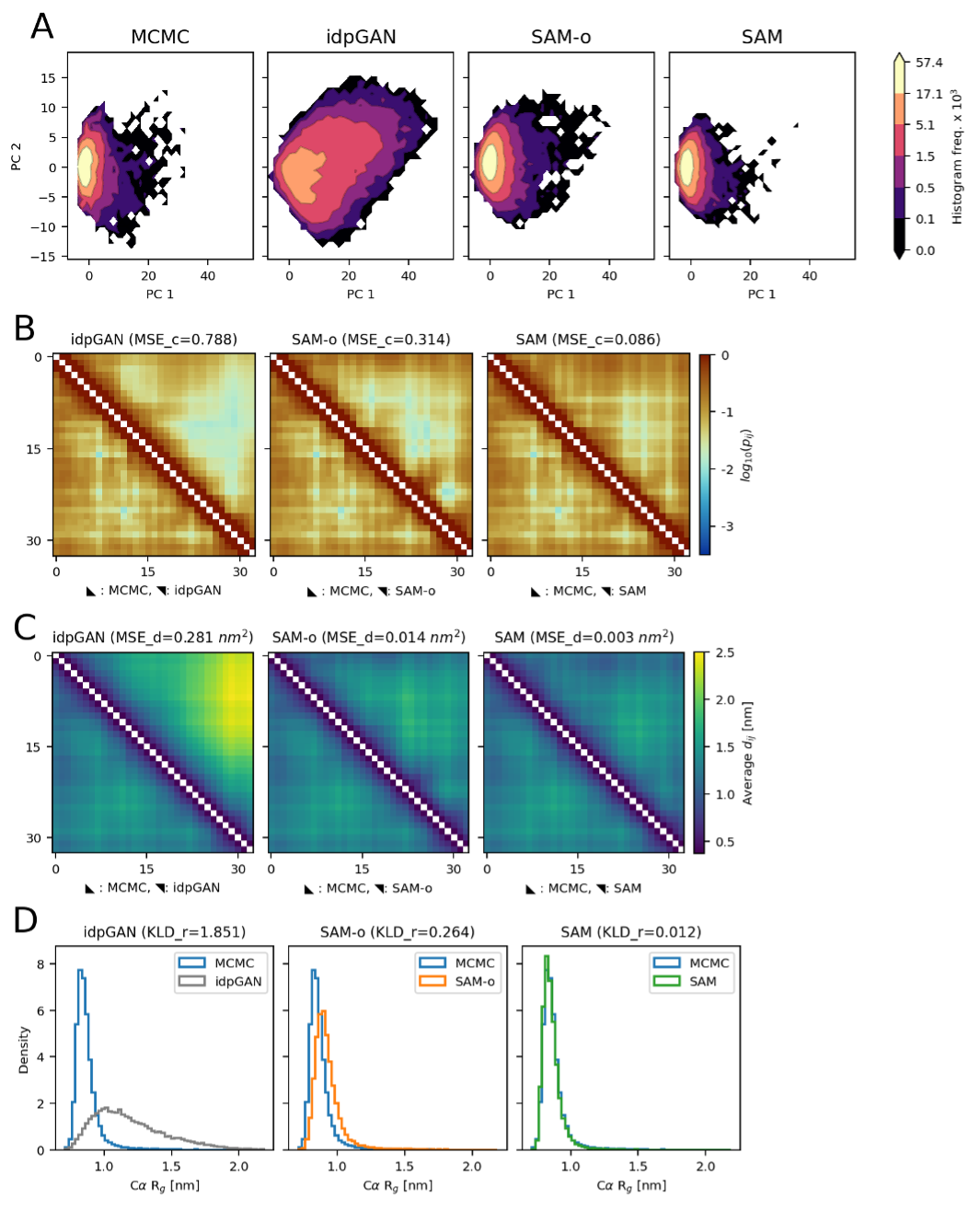

Supplement: S5 Fig — (A) PCA histograms for the structural ensembles of idpGAN, SAM-o and SAM-e. Here, SAM-o is a model trained with original training set of idpGAN (1089 IDRs), while SAM-e was trained with the expanded training set of this study (3,259 peptides) and is the model discussed in most of the main text. Frequency values in colorbars are multiplied by 1 × 10^3^. (B) Cα-Cα contact maps of the three methods. The MSE_c scores of the ensembles are reported in brackets. (C) Average Cα-Cα distance maps of the three methods, with their MSE_d scores reported in brackets. (D) Cα Rg histograms the three methods, with KLD_r values in brackets. (TIF) [file pcbi.1012144.s010.tif]

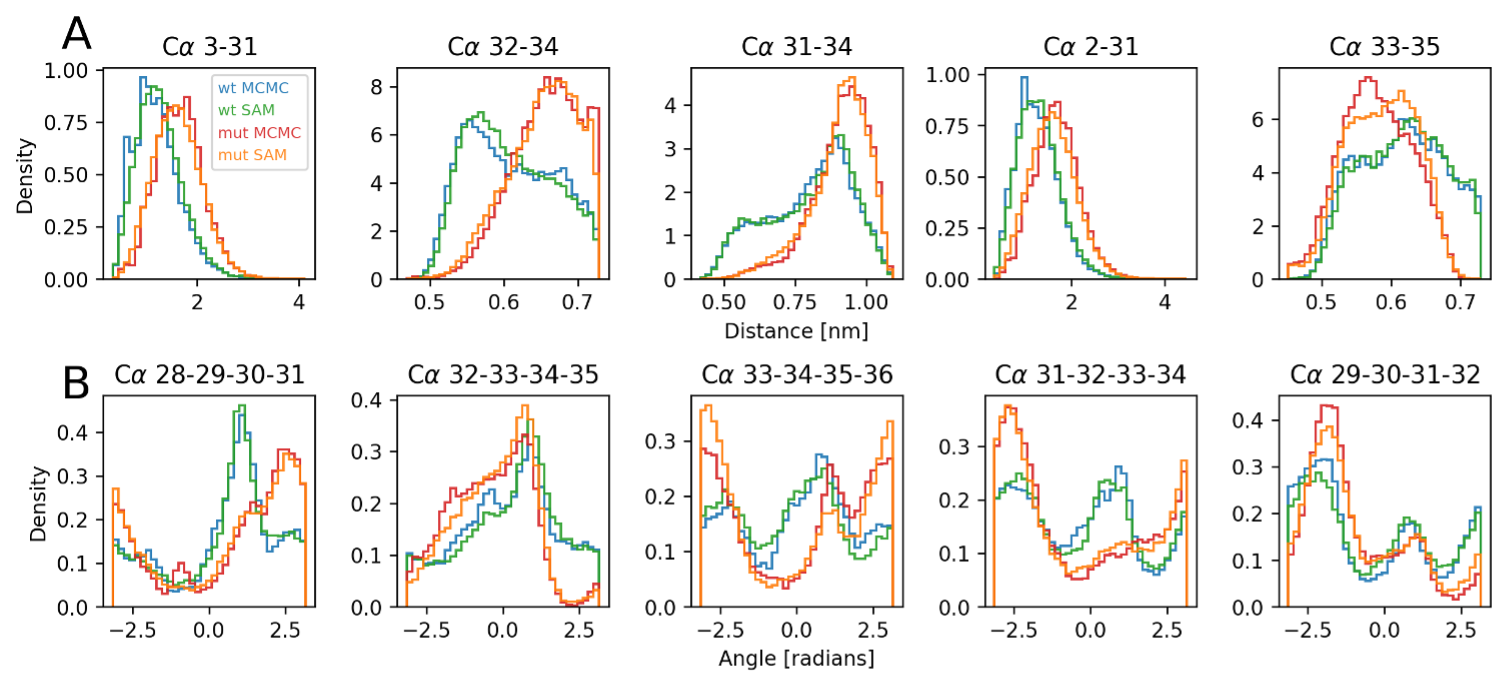

Supplement: S6 Fig — (A) Histograms of the five Cα-Cα distance distributions with the highest JS divergence between the wild-type and mutant ensembles from MCMC simulations. Residue indices of the Cα atoms are reported on top of the subplots (B). Histograms of the five α angle distributions with the highest JS divergence between the wild-type and mutant ensembles from MCMC. Residue indices of the Cα atoms defining the torsion angles are reported on top. (TIF) [file pcbi.1012144.s011.tif]

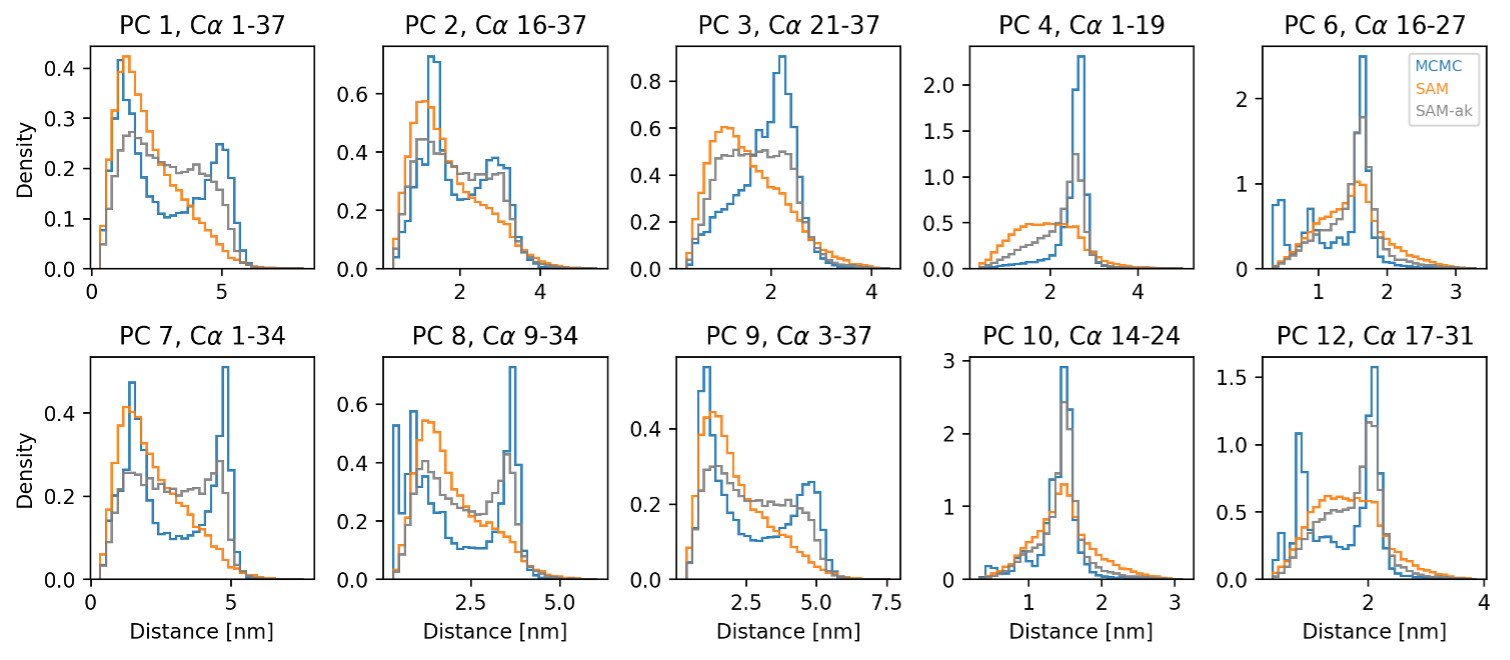

Supplement: S7 Fig — The plots show data for those Cα-Cα distances that exhibit the highest absolute loading value for 10 of the top principal components (PCs) identified in a PCA on the MCMC ensemble (refer to the main text for PCA details). The distances associated with PC 5 and 11 are not shown since it is the same shown for PC 1. SAM-ak was trained on the full training set of SAM and additional simulation data from three peptides with sequences similar to ak37. (TIF) [file pcbi.1012144.s012.tif]

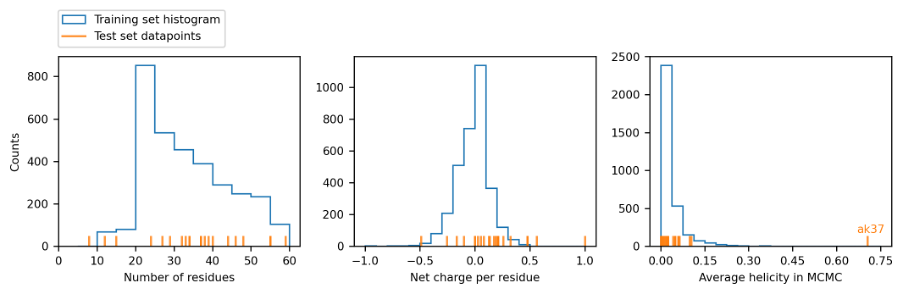

Supplement: S8 Fig — Left panel: lengths of the peptides. Central panel: net charge per residue. Right panel: average helicity in an ensemble of 10,000 conformations from MCMC simulations. Helicity is defined as the fraction of residues in an all-atom peptide conformation found in a helical state according to the DSSP algorithm [86]. The average helicity value of the ak37 peptide is highlighted in the plot. (TIF) [file pcbi.1012144.s013.tif]

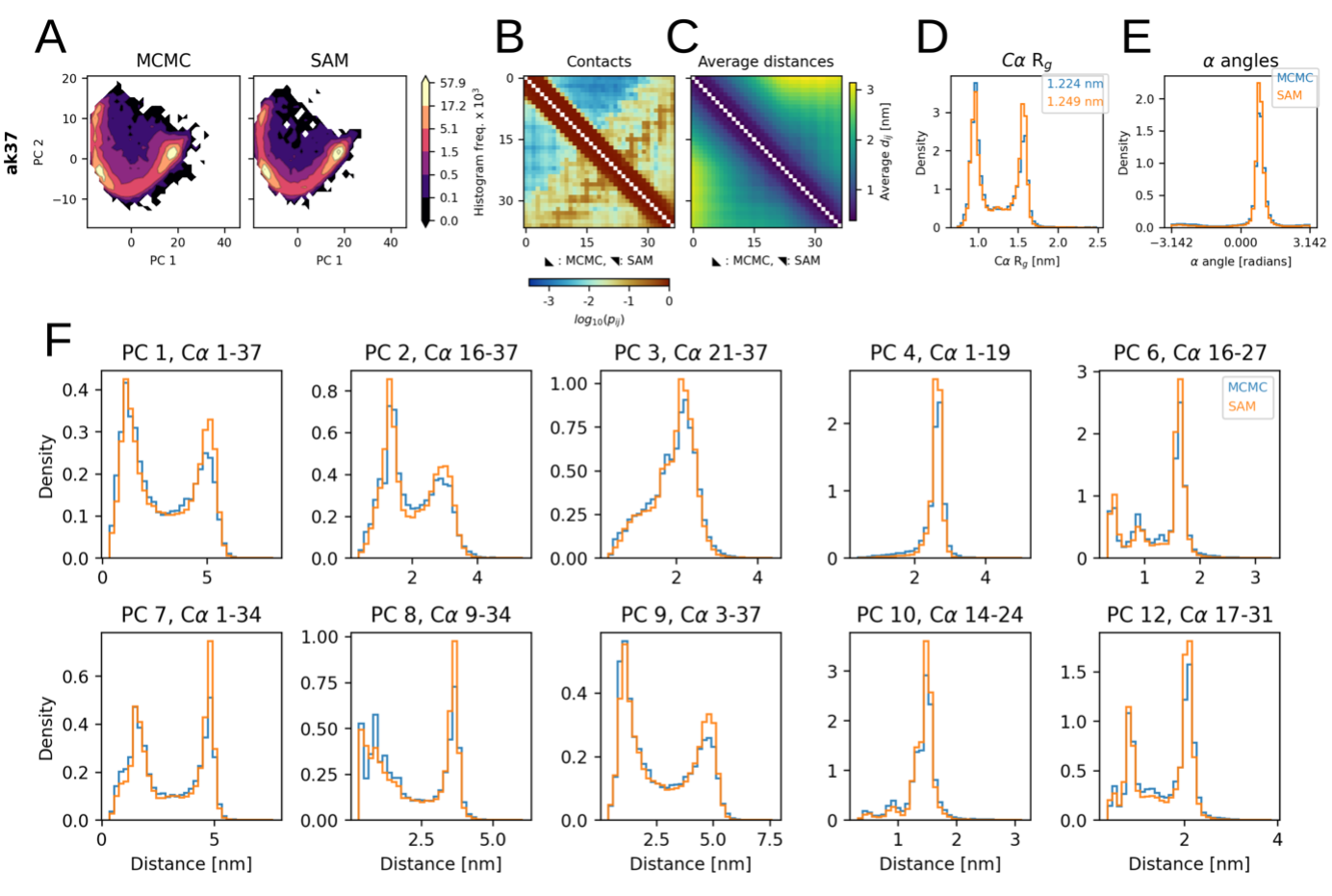

Supplement: S9 Fig — (A) to (E) Ensemble of ak37 modeled by a SAM version trained only on ak37 data. See Fig 2 in the main text for more details. (F) Cα-Cα distance distributions of the same ensemble. See S7 Fig for details. (TIF) [file pcbi.1012144.s014.tif]

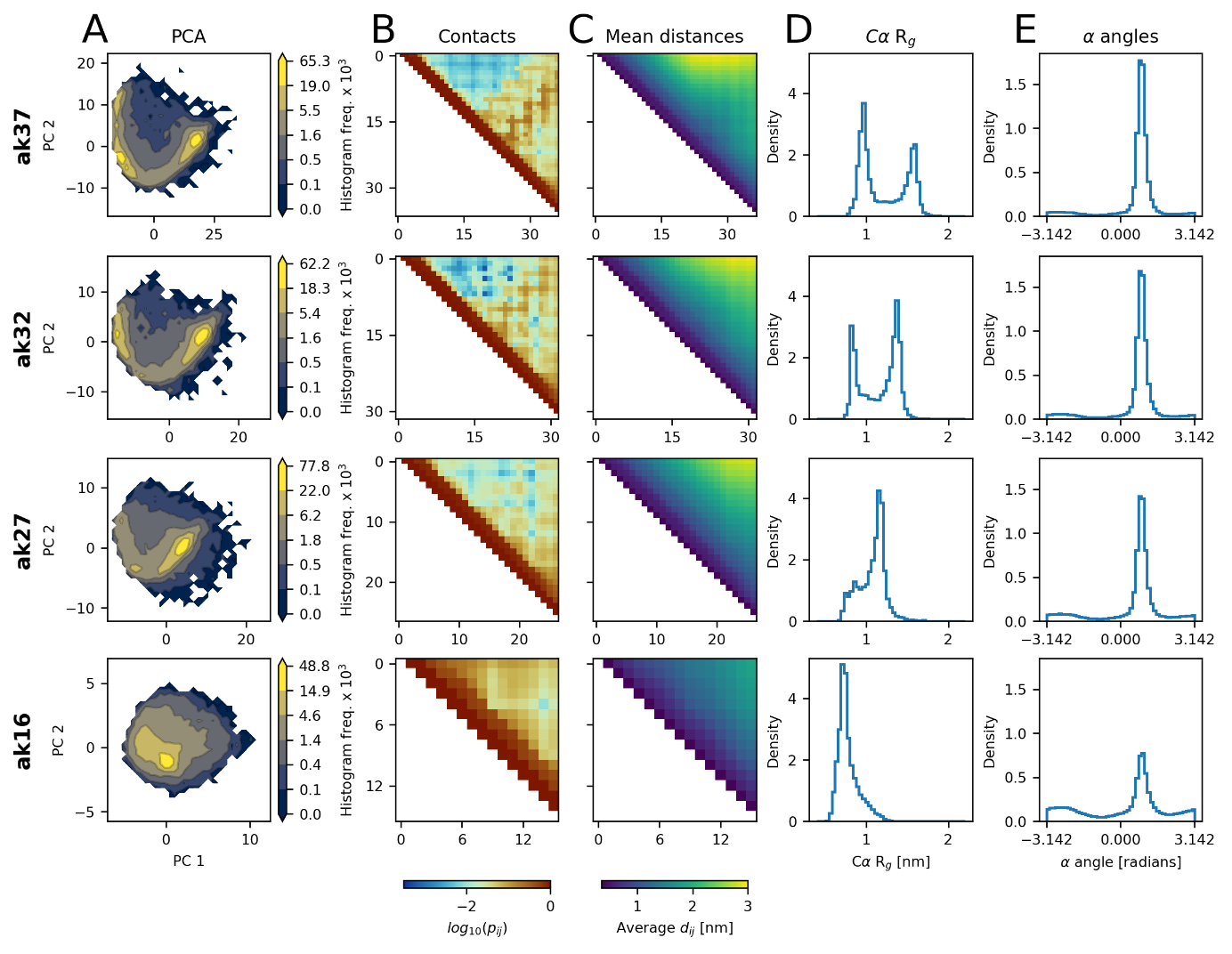

Supplement: S10 Fig — The ensembles consist of conformations randomly extracted from MCMC simulations. (A) PCA histograms for the four peptides. PCA was performed on each peptide independently and each row uses its own principal axes. (B) Cα-Cα contact maps. (C) Average Cα-Cα distances. (D) Cα Rg histograms. (E) α torsion angle histograms. (TIF) [file pcbi.1012144.s015.tif]

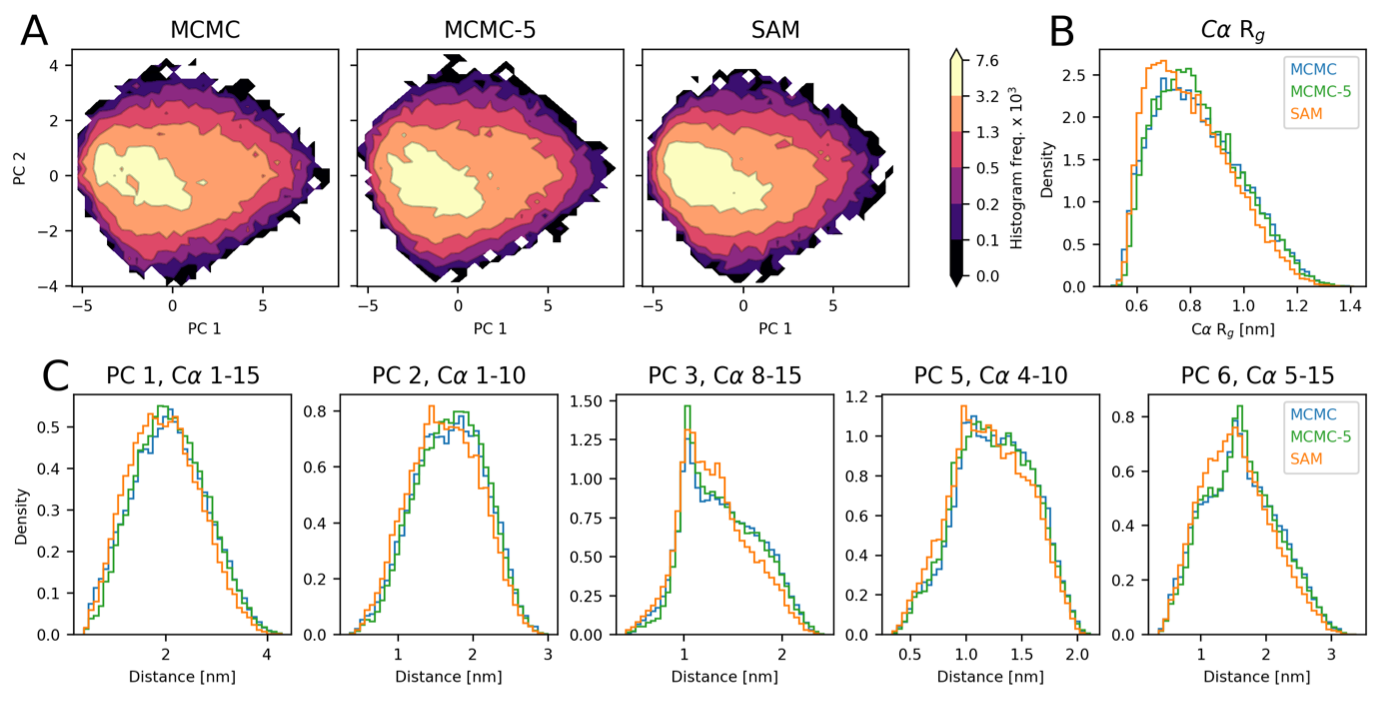

Supplement: S11 Fig — Refer to Fig 5 in the main text for a description of the panels. The ensemble from only 5 MCMC runs closely approximates the one from extensive sampling consisting of 73 MCMC runs (S2 Table). (TIF) [file pcbi.1012144.s016.tif]

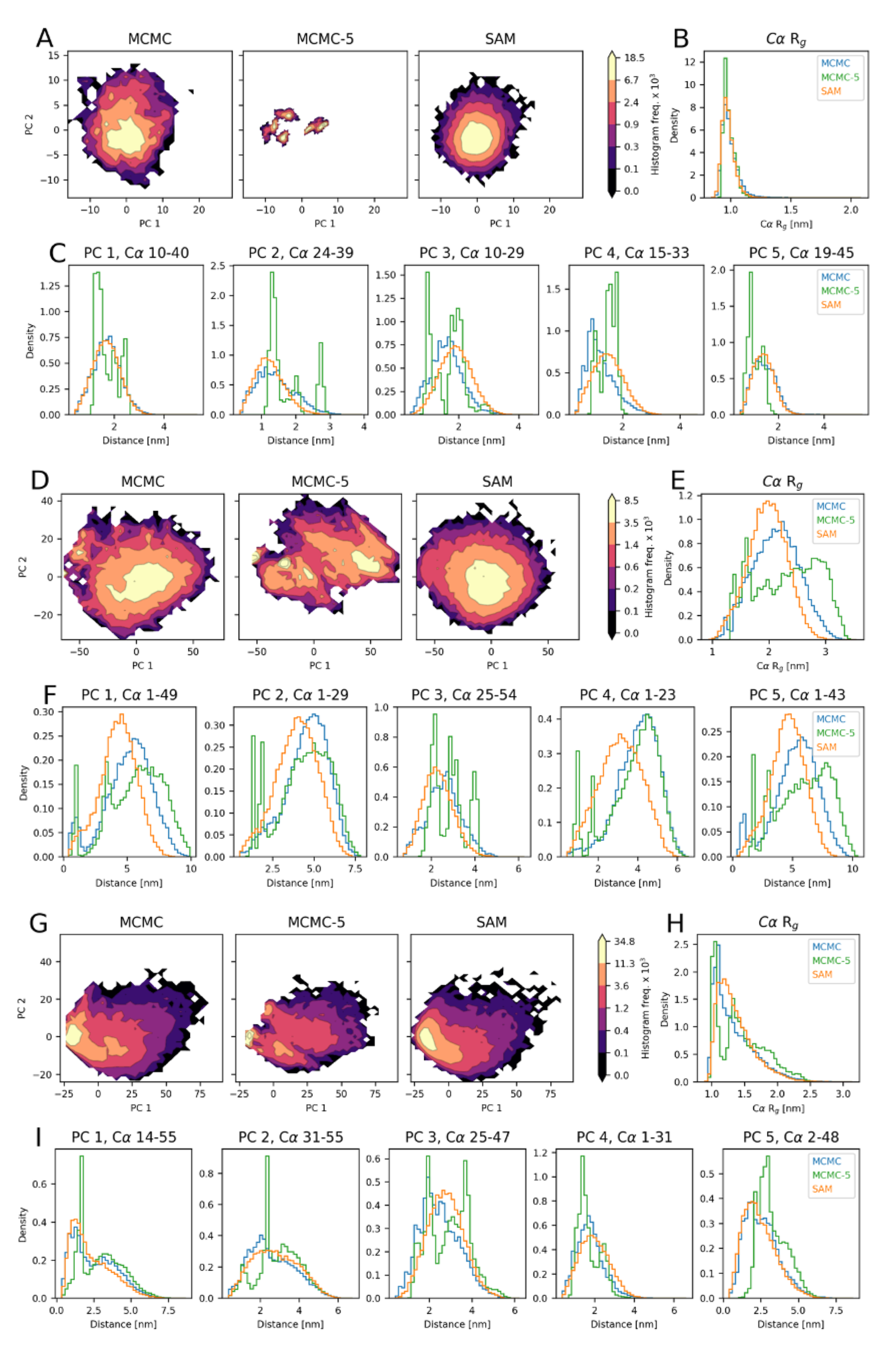

Supplement: S12 Fig — Refer to Fig 5 in the main text for a description of the panels. (A) to (C): data or nls. (D) to (F): data for protac. (G) to (I): data for protan. (TIF) [file pcbi.1012144.s017.tif]

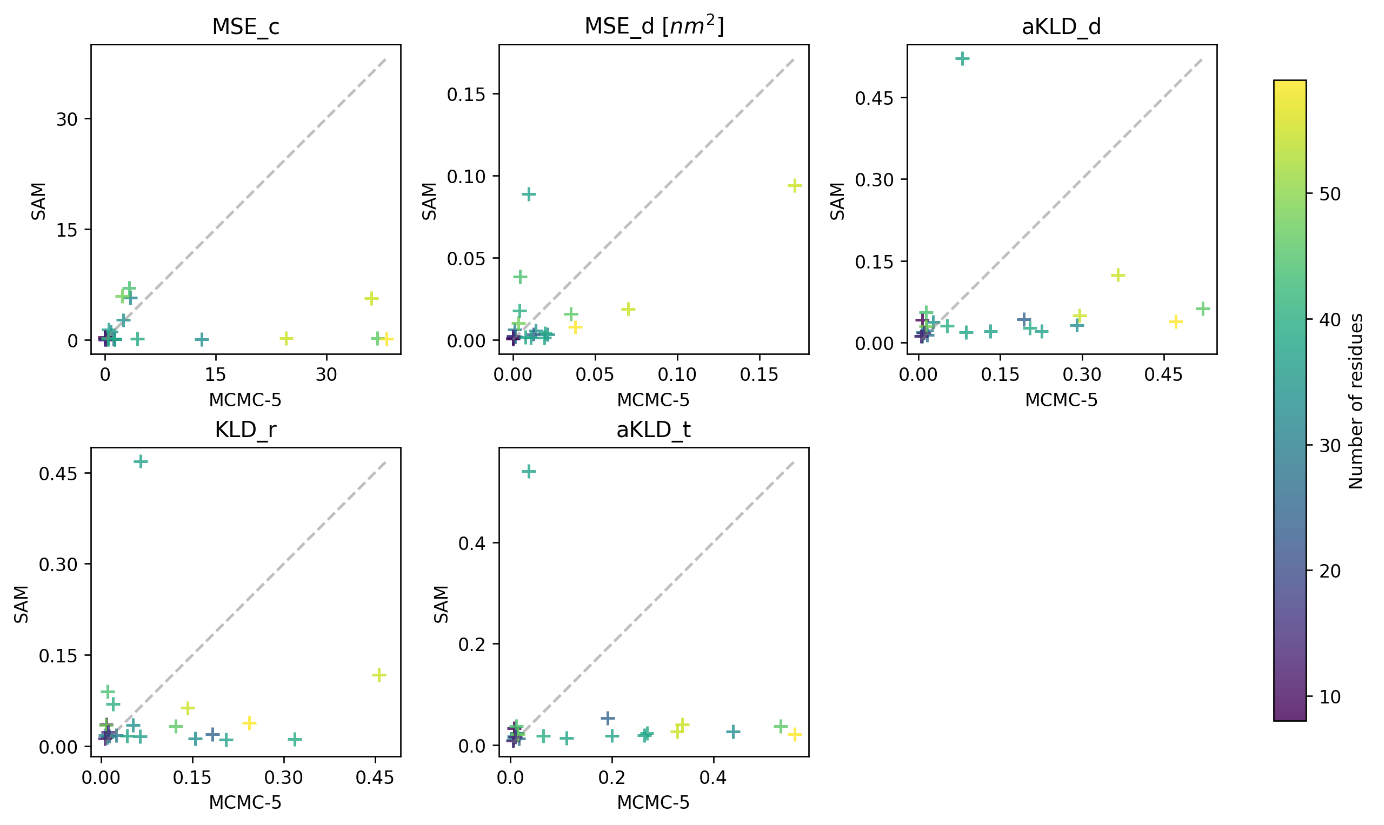

Supplement: S13 Fig — Each subplot confronts the evaluation scores of the two strategies for the 22 test set peptides. Markers are colored according to the length of the corresponding peptide. (TIF) [file pcbi.1012144.s018.tif]

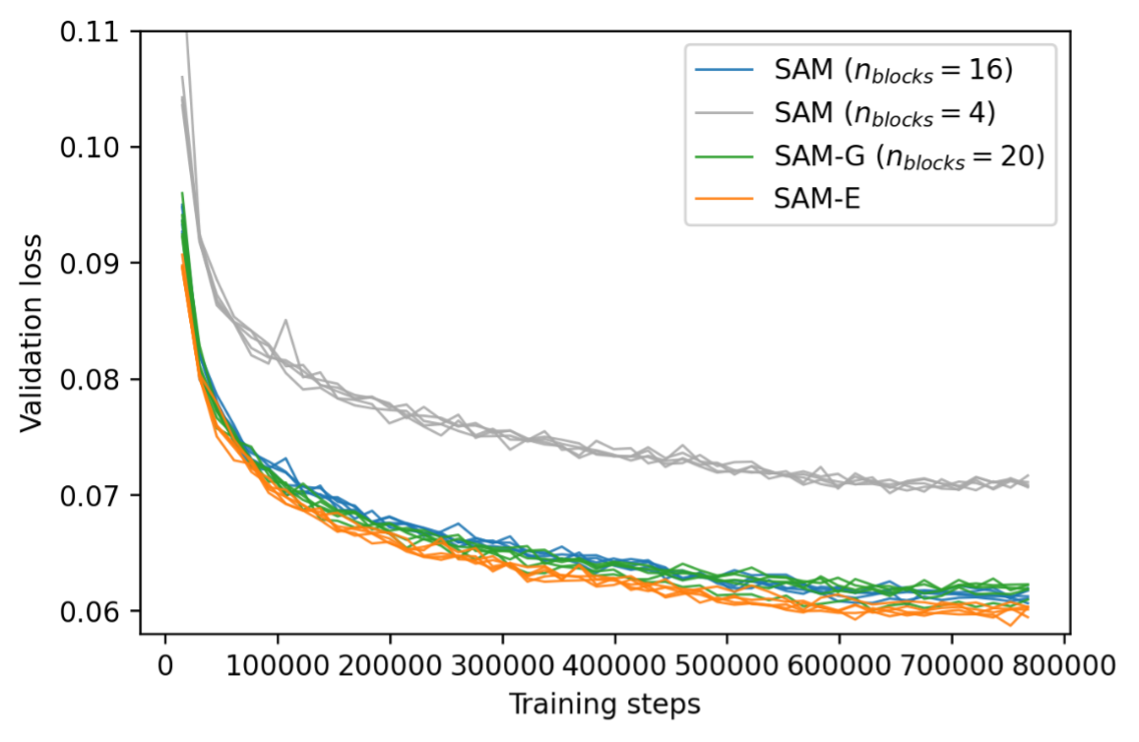

Supplement: S14 Fig — All the models with the “SAM” label have the same architecture for the noise prediction network, but have different number of transformer blocks in it. The SAM model with 20 blocks corresponds to the SAM-G model discussed in the main text. The SAM-E model has a different architecture, it has 16 blocks and incorporates 4 FrameDiff edge update operations. In case of the SAM models with 4 and 16 blocks, there is a large difference in validation loss at the end of training, which translates into a large difference in ensemble modeling performance (S4 Table). SAM-E has slightly better validation loss with respect to SAM versions with comparable numbers of layers, but it does not seem to significantly improve modeling performance (Table 1). For each model, we show validation loss curves from 5 different training runs. (TIF) [file pcbi.1012144.s019.tif]

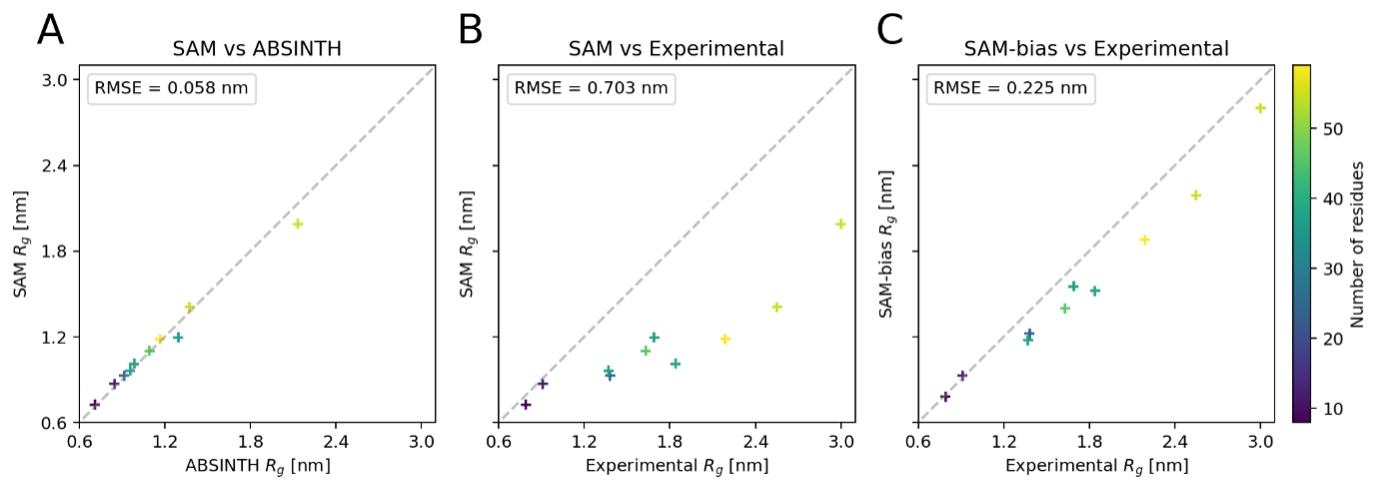

Supplement: S15 Fig — (A) Average Rg values from idpSAM and ABSINTH ensembles collected in our study. (B) Average Rg values from idpSAM and experimental measurements. (C) Average Rg values from idpSAM ensembles generated via the biased diffusion method and experimental measurements. (A) to (C) See S5 Table for more information on the ensembles. (TIF) [file pcbi.1012144.s020.tif]

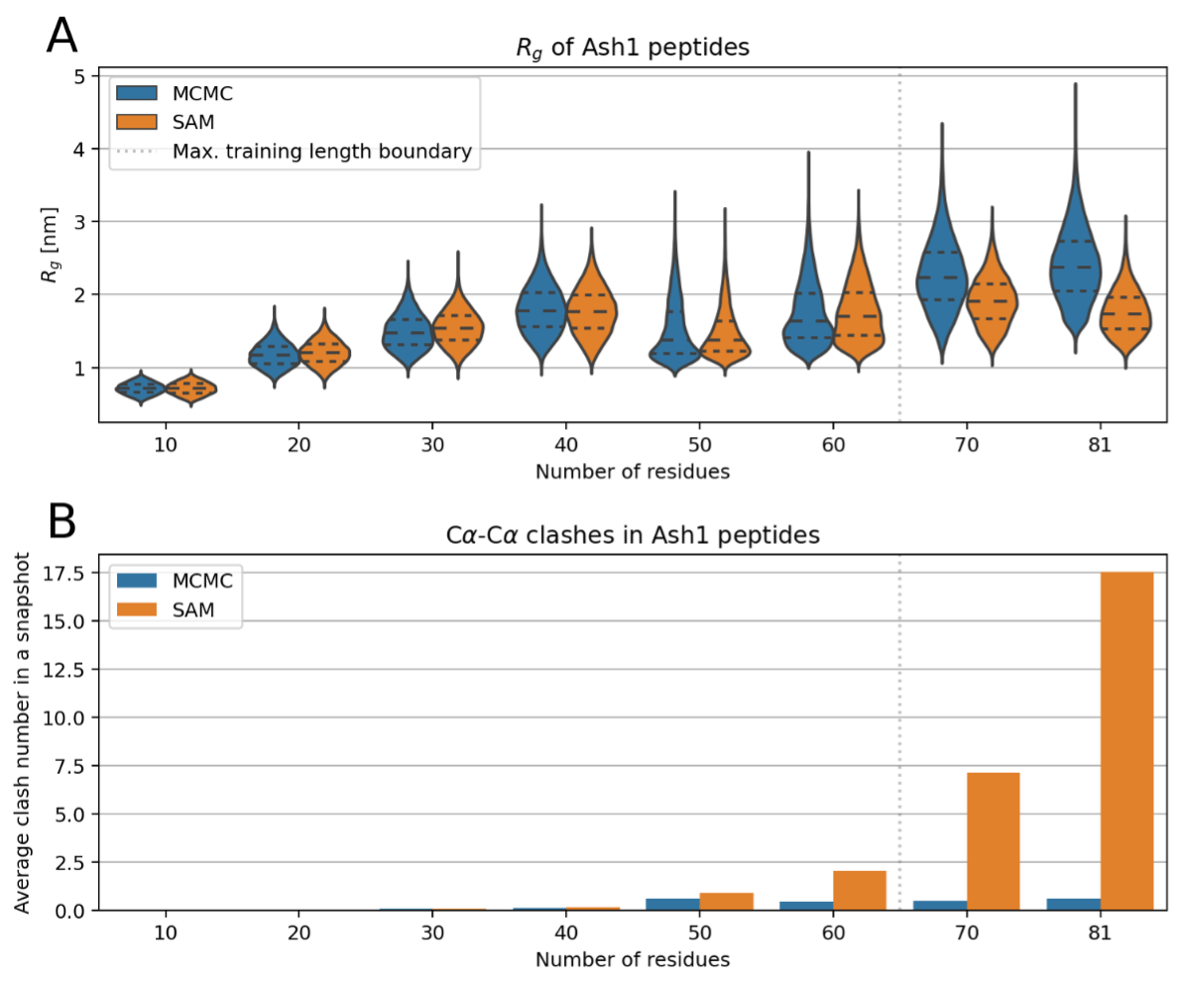

Supplement: S16 Fig — (A) Violin plots with Rg distributions of Ash1 peptides with different lengths in ABSINTH ensembles (blue) and idpSAM ensembles (orange). The dashes lines inside violins represent the quartiles of the datasets. All ensembles contain 10,000 snapshots. Data for ABSINTH simulations was collected by running 20 MCMC replicas at 298 K using the protocol described in the Methods. IdpSAM ensembles were converted to all-atoms via cg2all. (B) Average number of Cα-Cα clashes in snapshots of the same ensembles. Here, a clash is defined as a distance < 0.4 nm between two Cα atoms with sequence separation > 2. (TIF) [file pcbi.1012144.s021.tif]

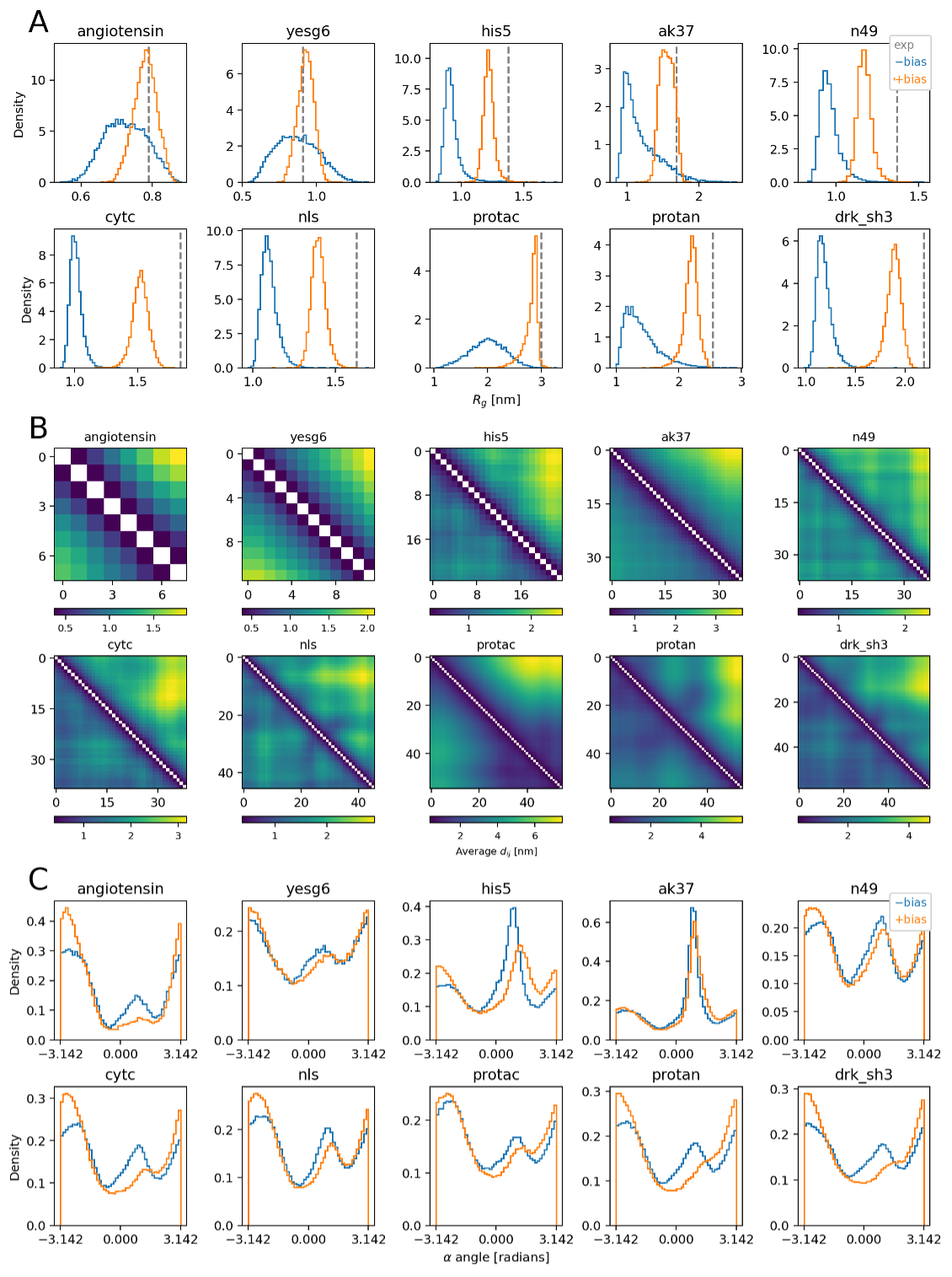

Supplement: S17 Fig — (A) Rg histograms of unbiased (blue) and biased (orange) idpSAM ensembles. Vertical gray lines are the experimental values. (B) Average Cα-Cα distances. Unbiased and biased ensembles are in the lower and upper triangles, respectively. (C) α torsion angle histograms. (A) to (C) See S5 Table for more information on the ensembles. (TIF) [file pcbi.1012144.s022.tif]

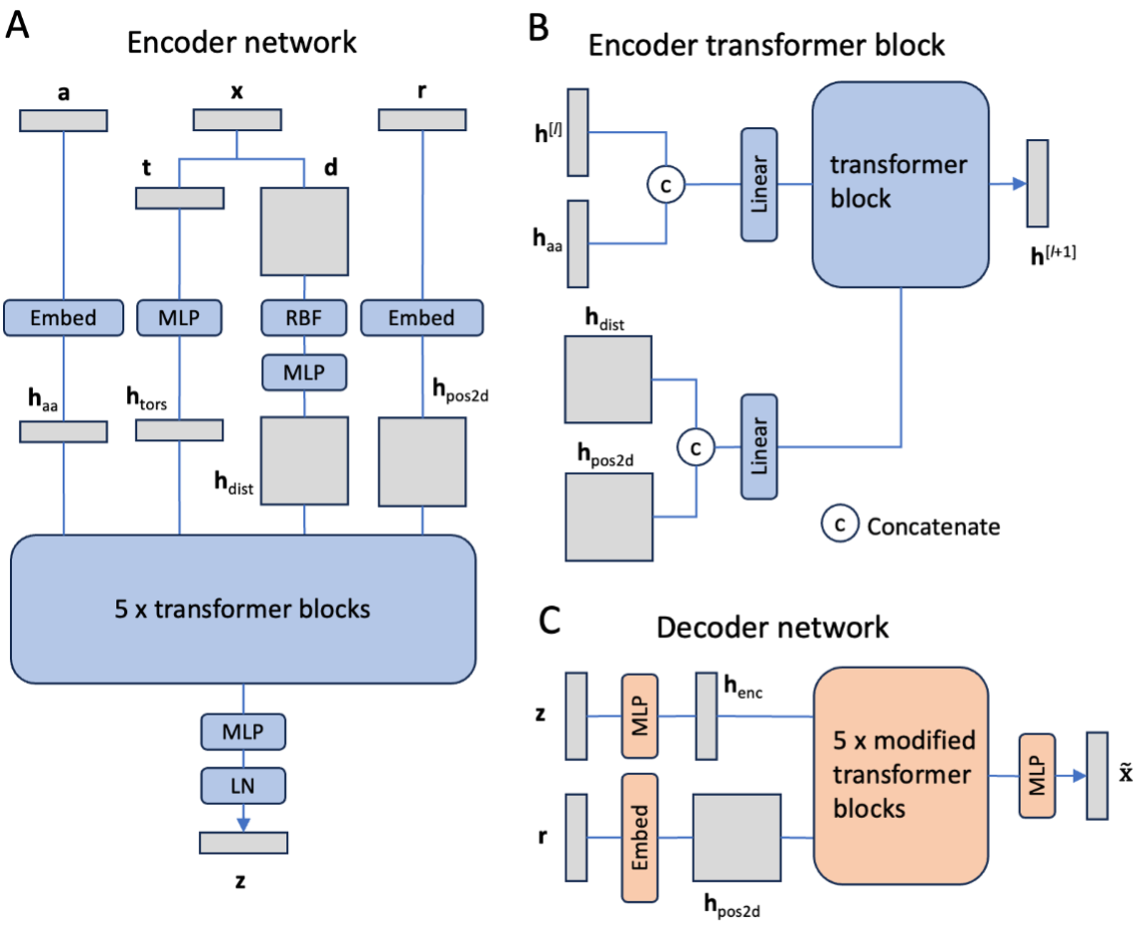

Supplement: S18 Fig — (A) Outline of the encoder network. The elements of the network are colored in blue, the tensors being processed by it are colored in gray. The tensor r represents the numerical indices of the input peptide, which are used to generate a 2d relative positional embedding h2dpos. Embed: embedding layer. MLP: multilayer perceptron. RBF: radial base function for embedding interatomic distances. LN: layer normalization. (B) Preparation of the input of a transformer block in the encoder. (C) Outline of the decoder network. (TIF) [file pcbi.1012144.s023.tif]

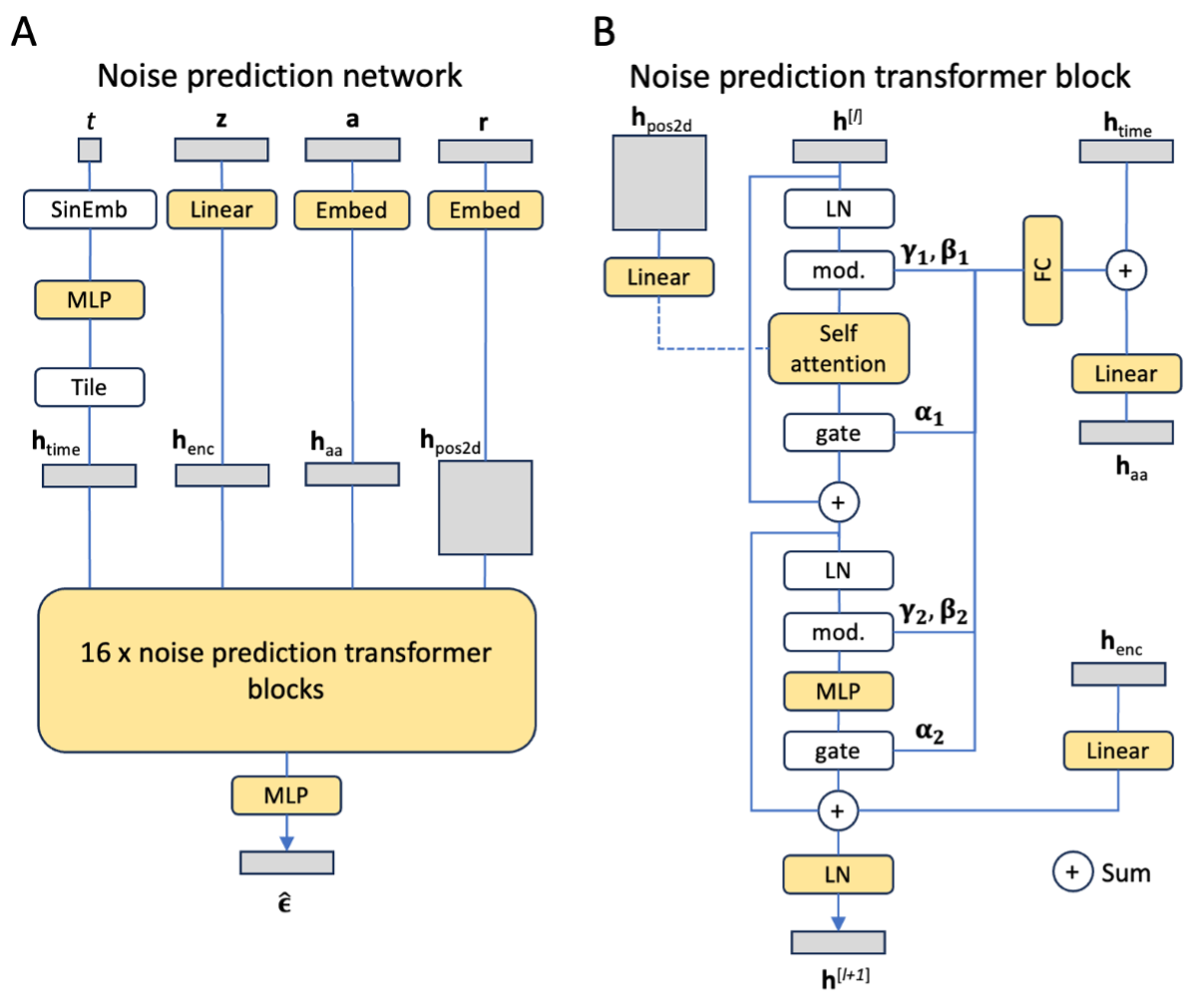

Supplement: S19 Fig — (A) Outline of the entire noise prediction network. SinEmb: sinusoidal embedding. (B) Illustration of a transformer block of the network. FC: fully-connected module consisting of an activation and a linear layer. LN: layer normalization (colored in white if does not have learnable elementwise affine parameters). Mod.: modulate operation for adaLN-Zero. Gate: gate operation of adaLN-Zero. (TIF) [file pcbi.1012144.s024.tif]

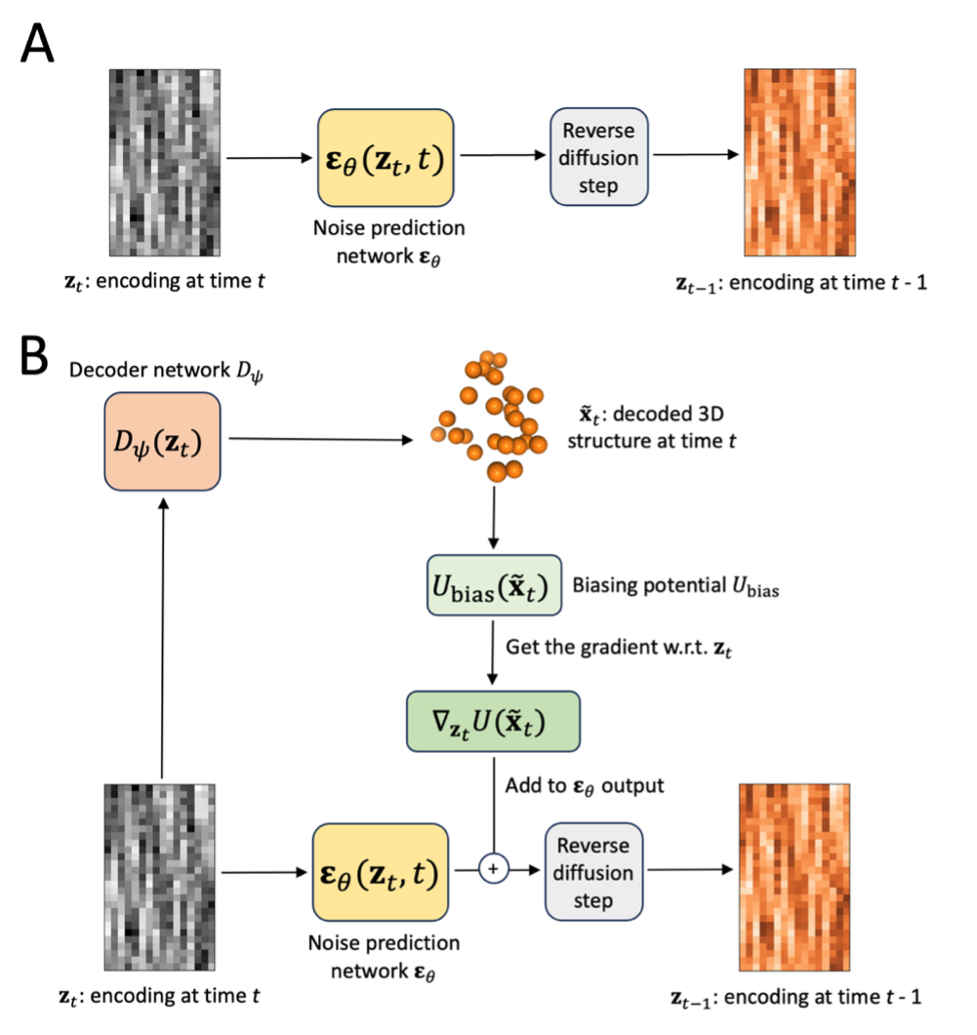

Supplement: S20 Fig — (A) Default step when sampling with a diffusion model. Only the output of the noise prediction network guides reverse diffusion. (B) Biased diffusion step. The output of the noise prediction network is summed to the gradient of a biasing potential. The biasing potential evaluates the 3D representation of an encoding at time t. The 3D representation is reconstructed from the encoding via the decoder network. The biasing potential can be used to guide diffusion to generate encodings representing 3D conformations with some target structural property, such as a certain Rg value. (TIF) [file pcbi.1012144.s025.tif]
