# Supplementary material for: Transferable deep generative modeling of intrinsically disordered protein conformations
Source: PLoS Comput Biol. 2024 May 23;20(5):e1012144. doi: 10.1371/journal.pcbi.1012144 (PMC11152266; doi:10.1371/journal.pcbi.1012144)
Supplement: S1 Table — (DOCX) [file pcbi.1012144.s026.docx]

**S1 Table. Reconstruction quality by the AE.**

| **Strategy** | $\boldsymbol{C}_{\boldsymbol{dist}}$ | $\boldsymbol{C}_{\boldsymbol{tors}}$ |
| --- | --- | --- |
| AE^a^ ($c=16$) | (3.64 ± 1.33) × 10^-3^ | (1.96 ± 0.69) × 10^-3^ |
| AE ($c=4$) | (4.13 ± 0.73) × 10^-2^ | (6.22 ± 1.06) × 10^-3^ |
| AE ($c=8$) | (7.34 ± 2.63) × 10^-3^ | (3.10 ± 0.86) × 10^-3^ |
| AE ($c=32$) | (2.49 ± 0.89) × 10^-3^ | (1.63 ± 0.59) × 10^-3^ |
| Perturbed^b^ (0.010 Å) | (6.91 ± 1.03) × 10^-4^ | (5.90 ± 0.10) × 10^-5^ |
| Perturbed (0.025 Å) | (4.30 ± 0.64) × 10^-3^ | (3.70 ± 0.08) × 10^-4^ |
| Perturbed (0.05 Å) | (1.72 ± 0.26) × 10^-2^ | (1.48 ± 0.03) × 10^-3^ |
| Perturbed (0.1 Å) | (6.89 ± 1.02) × 10^-2^ | (5.95 ± 0.13) × 10^-3^ |

For each test set peptide, 10,000 snapshots were randomly extracted from MCMC data and average $C_{dist}$ and $C_{tors}$ values were computed between these snapshots and their autoencoded or perturbed versions. The table reports the mean (with standard errors) of these average values for the 22 test peptides.

^a^MCMC conformations were encoded and decoded by AE of idpSAM. The AE of the default idpSAM version has an encoding dimension $c=16$.

^c^MCMC conformations in which Cα positions were perturbed by adding different levels of Gaussian random noise (standard deviations are reported in parentheses).
