# Supplementary material for: Transferable deep generative modeling of intrinsically disordered protein conformations
Source: PLoS Comput Biol. 2024 May 23;20(5):e1012144. doi: 10.1371/journal.pcbi.1012144 (PMC11152266; doi:10.1371/journal.pcbi.1012144)
Supplement: S2 Table — (DOCX) [file pcbi.1012144.s027.docx]

**S2 Table**. **Properties of the 22 test set peptides.**

| **Name** | **Sequence^a^** | **L^b^** | **q/L^c^** | **UniProt^d^** | **298 K runs^e^** | **RE runs^f^** |
| --- | --- | --- | --- | --- | --- | --- |
| angiotensin | DRVYIHPF | 8 | 0.00 | P01019 | 80 | - |
| yesg6 | YESGGGGGGATD | 12 | -0.17 | - | 90 | - |
| DP03125r003 | KPSNCQNKESASKQS | 15 | 0.13 | O14965 | 73 | - |
| his5 | DSHAKRHHGYKRKFHEKHHSHRGY | 24 | 0.21 | P15516 | 149 | - |
| Q9EP54 | MACYPVNIRARGLGKNMGMKSRGRGKG | 27 | 0.26 | **Q9EP54** | 150 | - |
| P02338_0 | MRSFDQGSTRAPARERCRRQRPEGRSAQR | 29 | 0.21 | **P02338** | 150 | - |
| Q91185 | MRRQASLPARRRRRVRRTRVVRRRRRVGRRRH | 32 | 0.56 | **Q91185** | 75 | - |
| Q2KXY0 | MFDNASTRNNKRERGKRQGKQTRTQRHADRSQT | 33 | 0.21 | **Q2KXY0** | 175 | - |
| P27205 | AGSKSRSRSRSRSRSKSPAKSASPKSAASPRASR | 34 | 0.32 | **P27205** | 150 | - |
| synthetic | RRRRRRRRRRRRRRRRRRRRRRRRRRRRRRRRRR | 34 | 1.00 | - | 60 | - |
| ak37 | AAKAAAAKAAAAKAAAAKAAAAKAAAAKAAAAKAAGY | 37 | 0.19 | - | 149 | - |
| cgrp_wt_fl | ACDTATCVTHRLAGLLSRSGGVVKNNFVPTNVGSKAF | 37 | 0.08 | **P06881**^*^ | 175 | - |
| cgrp_mt_fl | ACDTATCVTHRLAGLLSRSGGVVKNNFVPTDVGPWSF | 37 | 0.03 | **P06881**^*^ | 169 | - |
| n49 | GCQTSRGLFGNNNTNNINNSSSGMNNASAGLFGSKPFA | 38 | 0.05 | Q02199 | 194 | - |
| cytc_nter | MIFFMVMPIMIGGFGNWLVPLMIGAPDMAFPRMNNSFWL | 39 | 0.00 | P00395 | 184 | - |
| sic1_nterm_40 | MTPSTPPRSRGTRYLAQPSGNTSSSALMQGQKTPQKPSQN | 40 | 0.12 | P38634 | 158 | - |
| O13030 | MAYGRARSRGRSVRRRRRGRSPGRRRRGRRSDNDAPRRRRRRRQ | 44 | 0.48 | **O13030** | 75 | - |
| nls | ACETNKRKREQISTDNEAKMQIQEEKSPKKKRKKRSSKANKPPEFA | 46 | 0.17 | Q03281 | 184 | 69 |
| P83266 | ARRRHSMKKKRKSVRRRKTRKNQRKRKNSLGRSFKQHGFLKQPPRFRP | 48 | 0.48 | **P83266** | 65 | - |
| protac | CEEGGEEEEEEEEGDGEEEDGDEDEEAESATGKRAAEDDEDDDVDTKKQKTDEDC | 55 | -0.49 | P06454-2 | 137 | 56 |
| protan | CDAAVDTSSEITTKDLKEKKEVVEEAENGRDAPANGNANEENGEQEADNEVDEEC | 55 | -0.25 | P06454-2 | 137 | 59 |
| drk_sh3 | MEAIAKHDFSATADDELSFRKTQILKILNMEDDSNWYRAELDGKEGLIPSNYIEMKNHD | 59 | -0.10 | Q08012 | 166 | 69 |

^a^Positively charged residues are in blue, negatively charged in red.

^b^Number of residues in a peptide.

^c^Net charge per residue of a peptide.

^d^UniProt accession number of the sequence. Synthetic peptides have an empty value. Bold font corresponds to sequences covering their entire UniProt entry.

^e^Number of MCMC runs at 298 K.

^f^Number of replica exchange (RE) MCMC runs.

^*^Full sequence of the biologically-active peptide.
