# Supplementary material for: Transferable deep generative modeling of intrinsically disordered protein conformations
Source: PLoS Comput Biol. 2024 May 23;20(5):e1012144. doi: 10.1371/journal.pcbi.1012144 (PMC11152266; doi:10.1371/journal.pcbi.1012144)
Supplement: S3 Table — (DOCX) [file pcbi.1012144.s028.docx]

**S3 Table. Properties of the ak synthetic peptides.**

| **Name** | **Sequence^a^** | **L^b^** | **q/L^c^** | **298 K runs^d^** |
| --- | --- | --- | --- | --- |
| ak16 | YGCAKAAAAKACAAKA | 16 | 0.19 | 5 |
| ak27 | AAKAAAAKAAAAKAAAAKAAAAKAAGY | 27 | 0.19 | 5 |
| ak32 | AAKAAAAKAAAAKAAAAKAAAAKAAAAKAAGY | 32 | 0.19 | 5 |
| ak37 | AAKAAAAKAAAAKAAAAKAAAAKAAAAKAAAAKAAGY | 37 | 0.19 | 149 |

^a^Positively charged residues are in blue, negatively charged in red.

^b^Number of residues in a peptide.

^c^Net charge per residue of a peptide.

^d^Number of MCMC runs at 298 K.
