# Supplementary material for: Transferable deep generative modeling of intrinsically disordered protein conformations
Source: PLoS Comput Biol. 2024 May 23;20(5):e1012144. doi: 10.1371/journal.pcbi.1012144 (PMC11152266; doi:10.1371/journal.pcbi.1012144)
Supplement: S4 Table — (DOCX) [file pcbi.1012144.s029.docx]

**S4 Table. Evaluation scores of ensembles from modified versions of SAM.**

| **Strategy** | **MSE_c** | **MSE_d [nm^2^]** | **aKLD_d** | **KLD_r** | **aKLD_t** |
| --- | --- | --- | --- | --- | --- |
| SAM^a^ | 1.421 ± 0.499 | 0.015 ± 0.006 | 0.057 ± 0.023 | 0.053 ± 0.021 | 0.047 ± 0.024 |
| SAM-ak^b^ | 1.470 ± 0.479 | 0.013 ± 0.005 | 0.044 ± 0.008 | 0.051 ± 0.013 | 0.029 ± 0.005 |
| $n_{blocks}=4$^c^ | 2.649 ± 1.061* | 0.024 ± 0.008* | 0.092 ± 0.030* | 0.094 ± 0.025* | 0.075 ± 0.037* |
| $n_{blocks}=8$ | 1.786 ± 0.641 | 0.013 ± 0.003 | 0.067 ± 0.027* | 0.060 ± 0.018 | 0.060 ± 0.032* |
| no-lr-sched^d^ | 2.326 ± 1.061* | 0.021 ± 0.008* | 0.065 ± 0.021* | 0.092 ± 0.037* | 0.049 ± 0.015* |
| no-adalnzero^e^ | 3.926 ± 1.656 | 0.038 ± 0.015 | 0.093 ± 0.030 | 0.167 ± 0.064* | 0.056 ± 0.026 |
| no-input-inject^f^ | 1.373 ± 0.449 | 0.018 ± 0.010 | 0.063 ± 0.028 | 0.077 ± 0.037 | 0.057 ± 0.032* |
| $c=4$^g^ | 11.582 ± 6.616* | 0.423 ± 0.384* | 0.740 ± 0.56* | 1.062 ± 0.621* | 0.131 ± 0.037* |
| $c=8$ | 1.546 ± 0.464 | 0.015 ± 0.004 | 0.096 ± 0.035* | 0.138 ± 0.083 | 0.065 ± 0.016* |
| $c=32$ | 1.779 ± 0.603 | 0.018 ± 0.007 | 0.074 ± 0.031 | 0.068 ± 0.028 | 0.067 ± 0.036* |

Average scores are reported along with standard errors for the 22 test set peptides. Unless specified, all models were trained with the full training set of the default SAM version. Unless specified, all models use the same AE with only the DDPM being re-trained.

^a^Default idpSAM version.

^b^IdpSAM version with a DDPM trained with the full training set and three ak synthetic peptides.

^c^IdpSAM version with 4 transformer blocks in the noise prediction network of the DDPM.

^d^A “linear with warm up” learning rate schedule is not used to train the DDPM.

^e^The adaLN-zero mechanism is not used in the transformer blocks of the noise prediction network. Instead, time step and amino acid embeddings are projected to the same dimension of the node embeddings and added to them before the first layer normalization operation of the block.

^f^The initial node embedding is not injected at every block of the noise prediction network (**S19 Fig**).

^g^IdpSAM version with encoding dimension $c=4$. Both the AE and DDPM were re-trained.

^*^Asterisks denote a statistically significant difference (using a Wilcoxon signed-rank test with a significance level of 0.05) between the scores of a strategy and of the default idpSAM version.
