# Supplementary material for: Transferable deep generative modeling of intrinsically disordered protein conformations
Source: PLoS Comput Biol. 2024 May 23;20(5):e1012144. doi: 10.1371/journal.pcbi.1012144 (PMC11152266; doi:10.1371/journal.pcbi.1012144)
Supplement: S5 Table — (DOCX) [file pcbi.1012144.s030.docx]

**S5 Table. Comparison of idpSAM *R*_g_ with experimental values for 10 test set IDRs.**

| **Name** | **Method^a^** | **Reference^b^** | **Exp.^c^** | **MCMC^d^** | **SAM^e*^** | **SAM-b^f*^** | **SAM-b breaks^g^ (%)** |
| --- | --- | --- | --- | --- | --- | --- | --- |
| angiotensin | SAXS | Ohnishi et al., 2006 | 0.79 | 0.71 | 0.72 | 0.77 | 0.1 |
| yesg6 | SAXS | Ohnishi et al., 2006 | 0.91 | 0.85 | 0.87 | 0.91 | 0.0 |
| his5 | SAXS | Cragnell et al., 2016 | 1.38 | 0.91 | 0.93 | 1.20 | 0.1 |
| ak37 | SAXS | Kohn et al., 2004 | 1.69 | 1.29 | 1.19 | 1.54 | 0.3 |
| n49 | FRET | Fuertes et al., 2017 | 1.37 | 0.96 | 0.96 | 1.16 | 0.0 |
| cytc_nter | SAXS | Fuertes et al., 2017 | 1.84 | 0.98 | 1.01 | 1.50 | 2.4 |
| nls | FRET | Fuertes et al., 2017 | 1.63 | 1.09 | 1.10 | 1.39 | 0.7 |
| protac | FRET | Müller-Späth et al., 2010 | 3.00 | 2.13 | 1.99 | 2.79 | 33.2 |
| protan | FRET | Müller-Späth et al., 2010 | 2.55 | 1.37 | 1.41 | 2.13 | 31.4 |
| drk_sh3 | SAXS | Choy et al., 2002 | 2.19 | 1.16 | 1.18 | 1.84 | 15.4 |

All *R*_g_ values in the table are expressed in units of nm.

^a^Experimental methodology used to measure *R*_g_.

^b^Reference to the article with the *R*_g_ measurement (cited in the main text).

^c^Experimental value of *R*_g_.

^d^Average *R*_g_ in an ensemble with 10,000 snapshots from our ABSINTH data.

^e^Average *R*_g_ in an ensemble with 10,000 snapshots from idpSAM.

^f^Average *R*_g_ in an ensemble with 10,000 $\times$ (100 - breaks)/100 snapshots from idpSAM sampling with biased diffusion.

^g^Percentage of snapshots from a SAM-b ensemble containing a chain break (defined as a distance between two adjacent Cα atoms > 0.5 nm).

^*^All-atom details reconstructed with the cg2all method.
