# Supplementary material for: Transferable deep generative modeling of intrinsically disordered protein conformations
Source: PLoS Comput Biol. 2024 May 23;20(5):e1012144. doi: 10.1371/journal.pcbi.1012144 (PMC11152266; doi:10.1371/journal.pcbi.1012144)
Supplement: S6 Table — (DOCX) [file pcbi.1012144.s031.docx]

**S6 Table**. **Properties of the 25 validation set peptides.**

| **Name^a^** | **Sequence^b^** | **L^c^** | **q/L^d^** |
| --- | --- | --- | --- |
| A0A0G2JEB6 (2015-2035) | MPDHMRSSDYAADQSSSHAE | 20 | -0.15 |
| A0A452G813 (22-44) | IGDQPNDSYCYNSAKNSTVLQG | 22 | -0.05 |
| Q54WT6 (92-120) | RLIISAFKEIKFENQEKTKLTKFEKYQK | 28 | 0.14 |
| C6JUP1 (37-66) | KDKCEKYAVPVMRGKFYFSYQCTSKCHEG | 29 | 0.10 |
| P45198 (5-34) | QCNMKWGAEEEKKIELQAQGLQPDTLFSD | 29 | -0.10 |
| Q7NPQ7 (3-34) | QPSVKKICRNCKIIRRNRVVRVICTDPRHKQ | 31 | 0.29 |
| Q924W7 (711-744) | LPEVSYQFPKLDRPTKQMREAEERLKAIPQFCF | 33 | 0.03 |
| P54944 (19-54) | HYEYNPYPQQDVYDPYQMDRQPALERRIAALERQN | 35 | -0.06 |
| P72021 (34-72) | VIYHSQGLNALQMQNQDKNLEIRNDEYLELAKEFFDKA | 38 | -0.16 |
| P49525 (107-145) | ELVRAADMDAIEDDKVTIVGPDLKDMEEGKTYPWAMIF | 38 | -0.08 |
| A0FKN6 (23-62) | SNQDPIVDGMRLVEGDMLFDDGPLFTERNAVKYDQQLWP | 39 | -0.13 |
| B2GB76 (161-200) | NAVNLTDGLDGLVTGLATISFAAYLVLALVQGQTEVALF | 39 | -0.08 |
| A6W5T3 (77-117) | RRVGGSTYQVPIEVRPTRSTTLALRWLVGYARQRREKTMT | 40 | 0.18 |
| B8IMM7 (3-43) | TYQPSKLVRKRRHGFRARMATVGGRRVIAARRARGRKRLS | 40 | 0.38 |
| P30071 (285-326) | QKLSKSSRAWMREHLDDPFVKKAQKEGYRARAAYKLLEIQE | 41 | 0.02 |
| B9L6M9 (2-43) | RSLKKGPFVDDHLMKKVLKAKEEKNPKPIKTWSRRSTITPD | 41 | 0.17 |
| A3M851 (7-48) | ETPFISNNTIKKVNILVPIILSISDKEKFFNVLGHPTSNLF | 41 | 0.10 |
| A1S9H3 (305-347) | WEGDVYTMSAATREGTKELAEKLFDFIKSLPDEAAAADPDKE | 42 | -0.14 |
| Q9BKZ9 (61-103) | FKEAYEEEESREYVIENGVKRLVNQHYDSRGYSSAGRGQKGR | 42 | 0.00 |
| P70441 (91-134) | DPETDERLKKLGVSIREELLRPQEKSEQAEPPAAADTHEAGDQ | 43 | -0.14 |
| Q6IEU7 (245-289) | TIVTLFYGTLFCMYVRPPSEKSVEESKIIAVFYTFLSPMLNPLI | 44 | 0.00 |
| Q99PJ7 (274-318) | LLAIHQRTHTGEKPYTCLECSRRFRQRTALVIHQRIHTGEKPYP | 44 | 0.11 |
| B7IJW2 (1-48) | ELVTIEKGNIFFKSLEYKDLEEELLKKVSLYPNLEEYSKEEILTIET | 47 | -0.09 |
| A6UQD9 (114-161) | SFNLRGAVLANVSGNSQDQLQETIVDAIQSGEEKMLPGLGVLFEVIW | 47 | -0.13 |
| Q53RK8 (70-120) | SSDRDRDRDRDRDRDRDRDRDRERRRDDDSRRDRDRDRDRDRGDSSRRDR | 50 | 0.04 |

For all sequences, 3 MCMC runs were performed at 298K.

^a^UniProt accession number of the sequence with the starting and ending residue positions in the full sequence shown in parentheses.

^b^Positively charged residues are in blue, negatively charged residue in red.

^c^Number of residues.

^d^Net charge per residue.
