# Supplementary material for: Transferable deep generative modeling of intrinsically disordered protein conformations
Source: PLoS Comput Biol. 2024 May 23;20(5):e1012144. doi: 10.1371/journal.pcbi.1012144 (PMC11152266; doi:10.1371/journal.pcbi.1012144)
Supplement: S7 Table — (DOCX) [file pcbi.1012144.s032.docx]

**S7 Table. Details of SAM neural network training.**

| **Training process feature** | **Autoencoder** | **Diffusion model** |
| --- | --- | --- |
| *n*_systems_^a^ | 1,239 (“part 1” and “part 2” of the full training set, see **S1 Text**) | 3,259 (full training set) |
| *n*_frames_^b^ | 1,580 | 300 |
| Number of training epochs | 60 | 50 |
| Batch size | 64 | 64 |
| Number of training steps | ~1,838,000 | ~765,000 |
| Optimizer^c^ | Adam | Adam |
| Learning rate schedule | Milestone rate. Starts with lr=0.0005, multiplies the lr by 0.5 at epoch 10, 20, 30, 40 and 50. | Linear with warm up. Starts at lr=0.0, linearly warms up to lr=0.0005 in the first 10,000 steps, then linearly drops reaching lr=5.0$\times$10^-6^ after another 615,000 steps. Stays at the same value until the rest of training. |
| Time required for training^d^ | 1-2 days | 2-3 days |

^a^Number of different peptides used in the training set.

^b^Number of MCMC snapshots extracted from the simulation data of a single peptide during a training epoch.

^c^With the exception of the initial learning rate, we kept all other Adam parameters at their default PyTorch value.

^d^On a single NVIDIA RTX2080Ti GPU, using PyTorch 1.11.0 with automatic mixed precision training.
